# Youthful Brain-Derived Extracellular Vesicle-Loaded GelMA Hydrogel Promotes Scarless Wound Healing in Aged Skin by Modulating Senescence and Mitochondrial Function

**DOI:** 10.34133/research.0644

**Published:** 2025-03-28

**Authors:** Yuzhu Wu, Jiajie Mao, Yanyan Zhou, Gaoying Hong, Haiyan Wu, Zihe Hu, Xiaoyuan Huang, Jue Shi, Zhijian Xie, Yanhua Lan

**Affiliations:** ^1^ Stomatology Hospital, School of Stomatology, Zhejiang University School of Medicine, Zhejiang Provincial Clinical Research Center for Oral Diseases, Key Laboratory of Oral Biomedical Research of Zhejiang Province, Cancer Center of Zhejiang University, Engineering Research Center of Oral Biomaterials and Devices of Zhejiang Province, Hangzhou 310000, China.; ^2^ Department of Dentistry, Sir Run Run Shaw Hospital, School of Medicine, Zhejiang University, Hangzhou 310016, China.

## Abstract

Slow wound healing in the elderly has attracted much attention recently due to the associated infection risks and decreased longevity. The “brain–skin axis” theory suggests that abnormalities in the brain and nervous system can lead to skin degeneration because abnormal mental states, like chronic stress, can have negative physiological and functional effects on the skin through a variety of processes, resulting in delayed wound healing and accelerated skin aging. However, it remains unclear whether maintaining a youthful brain has beneficial effects on aged skin healing. In light of this, we identified youthful brain-derived extracellular vesicles (YBEVs) and created a composite GelMA hydrogel material that encourages scarless wound healing in aged skin. We found that YBEVs reduce the expression of senescence, senescence-associated secretory phenotypes, and inflammation-associated proteins, and even restore dysfunction in senescent cells. Furthermore, by encouraging collagen deposition, angiogenesis, epidermal and dermal regeneration, and folliculogenesis, we demonstrated that YBEV-containing composite hydrogels accelerated scarless wound healing in skin wounds of aged rats. The pro-repairing speed and effect of this composite hydrogel even matched that of young rats. Subsequent proteomic analysis revealed the presence of numerous proteins within YBEVs, some of which may play a role in the regulation of skin energy intake, particularly through oxidative phosphorylation and mitochondrial function. In conclusion, the findings suggest that maintaining a youthful brain could potentially alleviate skin aging, and the proposed YBEVs-GelMA hydrogel emerges as a promising strategy for addressing age-related impairments in skin healing.

## Introduction

The skin serves as a vital physical barrier that keeps harmful substances and pathogenic microbes out of the body [1]. The body is more vulnerable to infection and may experience a number of inflammatory reactions when skin damage leads to the breakdown of its physiological structure [2]. Quick wound healing is important, but as people age, their ability to heal wounds gradually declines [3]. In the elderly, wounds that do not heal properly must be treated because they increase the risk of infections, inflammation, and reduced life expectancy [4]. Therefore, discovering new regulatory functions and developing novel therapeutic approaches to support wound healing in the elderly are necessary.

The intricate relationship of the “brain–skin axis” has been described in the literature—with both considered to originate from the same germ layer [5]—in psychology, endocrinology, skin neurobiology, inflammation, immunology, and pharmacology [6]. The neuroendocrine networks have long been well recognized, especially with the discovery of corticotropin-releasing factor (CRF), which defines the upper regulatory arm of the hypothalamic–pituitary–adrenal (HPA) axis [7,8]. The skin has been identified as a neuroendocrine organ, expressing a variety of brain and pituitary hormones, as well as multiple neuropeptides, to regulate local homeostasis in response to stress [9]. In contrast, abnormal mental states such as stress can promote skin aging. On the other hand, adopting a balanced diet or engaging in regular physical exercise may partially delay the progression of structural brain changes associated with aging, such as brain atrophy [10]. However, it remains unclear whether cultivating a healthy, youth-like brain can promote the healing of skin wounds in older adults. While the tight regulatory crosstalk between brain and skin has been represented in cellular phenotypic alterations, the underlying mechanisms remain to be elucidated [11].

Senescent cells (SnCs) are an important feature of the aging process, and they gradually accumulate in the body with age [12]. The accumulation of SnCs in the skin is closely related to skin aging and plays an important role in various senescence-related physiological and pathological processes [13]. SnCs are distinguished by DNA damage, cell cycle arrest, metabolic alterations, mitochondrial dysfunction, and diminished autophagic activity, which collectively contribute to the compromised wound-healing capacity observed in aging tissues [14,15]. Among these features, mitochondrial function is especially important since it is the central link in cell biology [16]. Thus, timely supplementation of healthy mitochondria is necessary to delay senescence or reduce aging. Strategies targeting mitochondrial translocation hold the promise of therapeutic advances for senescent tissue repair. Mitochondrial transfer mechanisms include tunneling nanotubes, cellular fusion, cellular junction, and extracellular vesicles (EVs) [17,18]. Cells must be involved in the first 3 modes, which presents risks related to immunogenicity, aneuploidy, instability source, and difficult storage. EVs are a cell-free therapy strategy that can avoid these risks.

EVs are membrane-bound vesicles expelled from cells, body fluids, and tissue into the extracellular space and can carry materials (proteins, RNA, and DNA) from one cell to another. EVs from elderly subjects are mediators of the progressive deterioration of age-related tissue dysfunction over time [19,20]. In recent decades, EV therapies have shown promise in the field of aging [21]. One study found that EVs from young bovine serum or bone marrow contribute to a marked reduction in the senescence phenotype in vivo [22]. In recent years, the concept of tissue-derived EVs (Ti-EVs) has gained traction. Compared to cell-EVs, Ti-EVs more accurately reflect the microenvironment of tissues; Ti-EVs are regarded as substantial mediators of distal regulation in a variety of biological processes.

In terms of clinical feasibility, EVs are a promising cell-free therapeutic approach for tissue regeneration and repair [23], but their utility is restricted by the rapid clearance of bodily fluids and circulation, which makes it difficult to maintain the gradual release of EVs at a particular location. The duration of EVs’ action can be compensated for by combining EVs with hydrogels. Hydrogels filled with EVs can improve EV stability and assist in delivering EVs to the damaged location for long-term in situ release [24]. Recent studies have shown that EV-loaded hydrogels act as new bioactive factors in tissue regeneration and repair [25]. However, EV-loaded hydrogels remain poorly studied in aging skin, which may have fewer collagen fibers, scarce stem cells, and insufficient blood supply.

In this study, we hypothesized that brain-EVs, as a novel paradigm, regulate aging fibrocyte metabolism and functions by delivering mitochondrion-related proteins. GelMA hydrogels containing EVs generated from youthful brains (YBEVs) were used to repair aging skin damage, and the results revealed that the YBEVs-GelMA hydrogels accelerated wound healing at the same pace as that of the young. Considering reversing brain aging through systemic interventions such as exercise and balanced diet, it is further confirmed that life improvement might delay skin aging and accelerate skin healing through the brain–skin axis. Our study also suggests that local application of GelMA hydrogels containing EVs with healthy mitochondrion-related proteins could be used as a potential therapeutic strategy to accelerate healing in aging skin.

## Results and Discussion

### Characterization and the anti-aging effect of YBEVs

**Fig. 1.**
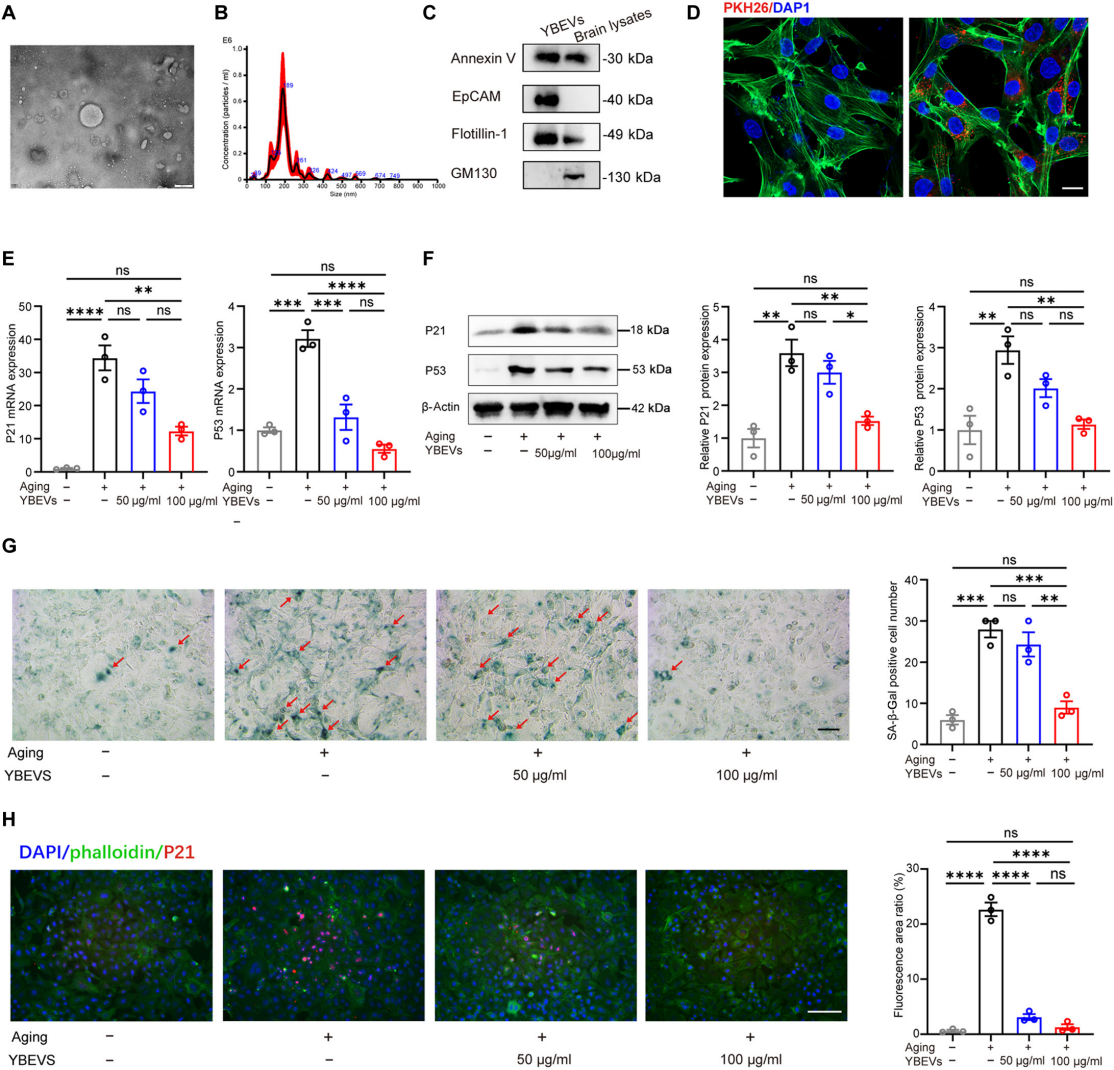
Characterization of YBEVs and their role in mitigating cellular senescence in HDFs. (A) TEM image of YBEVs. Scale bar, 200 nm. (B) YBEV size distribution was determined via nanoparticle tracking analysis. (C) WB analysis of annexin V, EpCAM, flotillin-1, and GM130 protein expression of YBEVs and brain lysates. (D) PKH26-labeled YBEVs were internalized by HDFs. Scale bar, 10 μm. (E) RT-qPCR analysis of senescence markers (P21 and P53) in HDFs incubated with H_2_O_2_ with or without YBEVs. (F) WB analysis of P21 and P53 protein expression of HDFs incubated with H_2_O_2_ with or without YBEVs. (G) Representative images of SA-β-Gal staining of HDFs incubated with H_2_O_2_ with or without YBEVs and quantitative analysis. Scale bar, 100 μm. (H) Representative immunofluorescence images of P21 and quantitative analysis of P21. Scale bar, 100 μm. All data are presented as means ± SEM; *n* = 3. *P < 0.05; **P < 0.01; ***P < 0.001; ****P < 0.0001; ns, *P* > 0.05.

In this study, YBEVs were extracted and identified. The results of the transmission electron microscopy (TEM) analysis showed that the shape of the particle was approximately elliptical, with a diameter of around 200 nm (Fig. 1A). We used a Nanosight NS300 instrument to determine the particle size of YBEVs to be approximately 189 nm (Fig. 1B). Moreover, EV-specific markers, such as annexin V and EpCAM, were highly enriched, as was flotillin-1. GM130 was at higher levels in the brain lysates but was not expressed in the EVs (Fig. 1C). These results suggest that the YBEVs isolated in this study met the MISEV2023 identification standards [26]. It is well known that EVs act classically through endocytosis by receptor cells [27]. Therefore, we first determined whether the YBEVs could be transferred to human dermal fibroblasts (HDFs) by tracking them with the fluorescent dye PKH26. After incubation for 8 h, red fluorescent PKH26-labeled YBEVs crossed the cell membrane into the cytoplasm of HDFs and accumulated around the nucleus (Fig. 1D). At the concentrations of 50 and 100 μg/ml used, YBEVs did not affect HDFs’ viability and even promoted cell proliferation (Fig. S1A).

Skin aging is closely related to oxidative stress in dermal fibroblasts. Oxidative damage in dermal fibroblasts can be studied as a model of dermal fibroblast aging, and H_2_O_2_ is a classical inducer of oxidative stress [28]. In this study, to determine the effect of the YBEVs on the aging effect of dermal fibroblasts, HDFs were cultured in the presence or absence of YBEVs (Fig. S1B). To further explore the senescence status of YBEVs on senescent fibroblasts, the expression of P53 and P21 in HDFs from different groups was detected using real-time quantitative polymerase chain reaction (RT-qPCR) and Western blotting (WB). As shown in Fig. 1E and F, YBEVs acted in a concentration-dependent manner, with both 50 and 100 μg/ml of YBEVs decreasing the expression of P53 and P21, and the EVs of the latter concentration resulted in a greater decrease in senescent markers, suggesting that YBEVs prevented cellular senescence.

Senescence-associated β-galactosidase (SA-β-Gal) is a β-galactosidase that exhibits increased activity during cellular senescence [29]. By detecting the activity level of SA-β-Gal in cells, the degree of cellular senescence can be assessed. Therefore, SA-β-Gal staining was used to investigate the effect of the YBEVs on senescent HDFs. H_2_O_2_ stimulation significantly increased the number of SnCs (green). YBEVs at concentrations of 100 μg/ml protected the cells from senescence, but a lower concentration of YBEVs (50 μg/ml) did not have the same effect (Fig. 1G). Consistent with the RT-qPCR and WB results, P21 immunofluorescence staining was strongest in H_2_O_2_-stimulated cells, whereas YBEVs significantly inhibited the expression of P21, and the 100 μg/ml concentration of YBEVs had a stronger anti-aging effect (Fig. 1H). Overall, under the classical H_2_O_2_ in vitro aging model, YBEVs could significantly decrease HDFs’ expression of senescence markers. They essentially demonstrated concentration dependence—the higher the number of YBEVs, the better the anti-aging effect—and did not show drug-like toxic side effects at high concentrations.

### YBEVs restore dysfunctional senescent HDFs

**Fig. 2.**
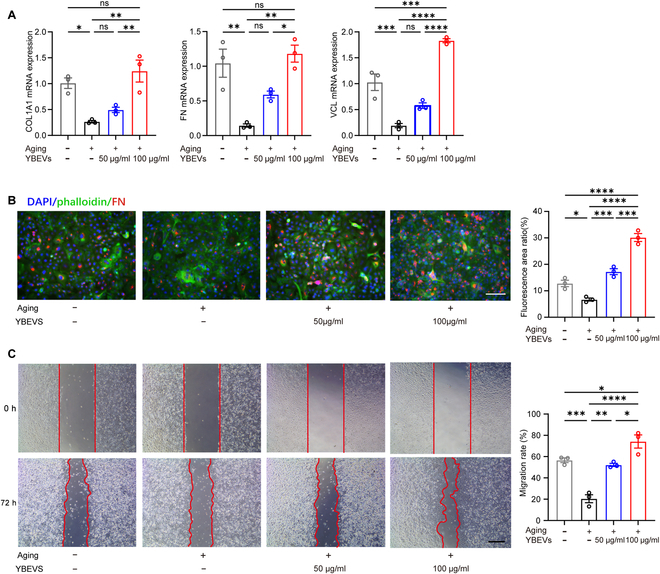
YBEVs restore dysfunctional HDFs. (A) RT-qPCR analysis of COL1A1, FN, and VCL markers in HDFs incubated with H_2_O_2_ with or without YBEVs. (B) Representative immunofluorescence images and quantitative analysis of FN. Scale bar, 100 μm. (C) Cell horizontal migration by scratch test and quantitative analysis (72 h). Scale bar, 200 μm. All data are presented as means ± SEM; *n* = 3. *P < 0.05; **P < 0.01; ***P < 0.001; ****P < 0.0001; ns, *P* > 0.05.

We previously explored the effect of YBEVs on the senescence phenotype of HDFs, and we wondered whether this senescence reduction could improve their function. To further explore the healing ability of YBEVs on senescent fibroblasts, collagen type 1 alpha 1 (COL1A1), fibronectin (FN), and vinculin (VCL) were used to characterize the cells. COL1A1 is a gene encoding collagen I, which acts as a marker of fibroblasts and promotes wound healing by promoting collagen synthesis and deposition [30], and adhesion-associated proteins like FN and VCL contribute to cell migration and proliferation [31–33]. The aging state decreases the expression of COL1A1, FN, and VCL. YBEVs at concentrations of both 50 and 100 μg/ml increased the expression of genes, which indicated that YBEVs promoted the ability of HDFs to form soft tissue and promote wound healing. YBEVs at concentrations of 100 μg/ml demonstrated a more efficient effect on the expression of COL1A1, FN, and VCL than 50 μg/ml (Fig. 2A). As shown in Fig. 2B, H_2_O_2_ stimulation significantly decreased the fluorescence for FN (red), and both YBEVs at concentrations of 50 and 100 μg /ml improved the expression of the cells. The capacity for cell migration was determined using scratch wound healing. The migratory ability of the HDFs was impaired by H_2_O_2_ (Fig. S2), but the migratory ability was significantly increased after adding the YBEVs, and the YBEVs at concentrations of 100 μg/ml were more efficient (Fig. 2C). Overall, YBEVs positively regulated functionally impaired HDFs and promoted soft tissue formation and the wound-healing ability of the cells.

### Synthesis and characterization of YBEVs-GelMA hydrogel

**Fig. 3.**
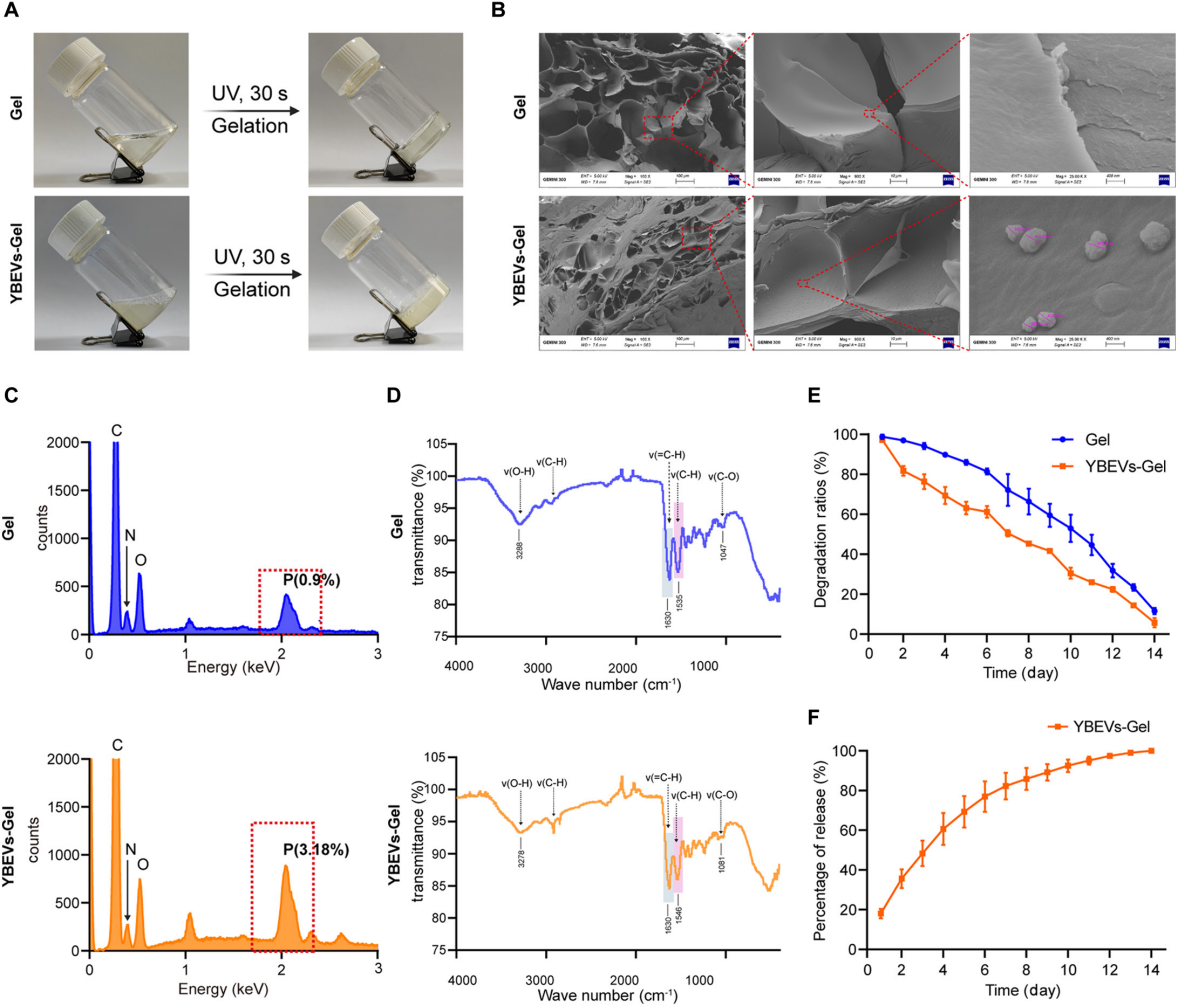
Characterization of GelMA hydrogel and YBEVs-GelMA hydrogel. (A) Photopolymerizable ability of GelMA hydrogel and YBEVs-GelMA hydrogel. (B) Scanning electron microscopy images for GelMA hydrogel and YBEVs-GelMA hydrogel. Scale bars, 100 μm, 10 μm, and 400 nm. (C) Energy-dispersive spectrometer analysis of GelMA hydrogel and YBEVs-GelMA hydrogel. (D) Fourier-transform infrared spectrometer spectra of GelMA hydrogel and YBEVs-GelMA hydrogel separately. (E) Degradation rates of GelMA hydrogel and YBEVs-GelMA hydrogel. (F) Release of YBEVs from YBEVs-GelMA hydrogel.

To further explore the role of YBEVs in vivo, we constructed GelMA hydrogel systems containing YBEVs to meet the advantages of slow and sustained release in vivo, nontoxicity, and good handling. First, we performed a series of characterizations of the hydrogels. GelMA hydrogel and YBEVs-GelMA hydrogel both existed as liquids at room temperature, and they were converted into hydrogel form when exposed to ultraviolet (UV) irradiation, which suggested that both possessed the ability of photopolymerization (Fig. 3A). More importantly, this good post-light plasticity allows for the expansion of skin repair scenarios. YBEVs-GelMA hydrogel could have good application potential on irregular traumatized surfaces.

We found that GelMA hydrogel has a typical loose and porous structure. In YBEVs-GelMA hydrogel, the internal structure is not as homogeneous as that of GelMA hydrogel due to the presence of YBEVs, but the YBEVs still show a dispersed distribution (Fig. 3B). Energy-dispersive x-ray spectroscopy (EDS) results suggested that both GelMA hydrogel and YBEVs-GelMA hydrogel contained C, N, O, and P elements. Compared to GelMA hydrogel, there were more P elements in YBEVs-GelMA hydrogel, with 3.18%. P is one of the characteristic elements of the phospholipid bilayer, which is the major component in the lipid bilayer of EVs, indicating that YBEVs-GelMA hydrogel contains YBEVs (Fig. 3C).

Fourier-transform infrared spectrophotometry was used to test the molecular structures of the GelMA hydrogel and YBEVs-GelMA hydrogel. The Fourier spectra of the 2 groups overlapped considerably, and their peaks were not substantially different (Fig. 3D). The results of the degradation experiments showed that the degradation of the hydrogel increased with the incubation time, and the GelMA hydrogel and YBEVs-GelMA hydrogel were almost completely degraded on day 14 (Fig. 3E). More importantly, we showed that YBEV release was continuous for 14 d, and the absolute majority of loaded YBEVs were released from the GelMA hydrogel (Fig. 3F). These results demonstrate the success of the YBEVs-GelMA hydrogel formulation and the sustained release of YBEVs to maintain adequate concentrations in skin wounds.

### YBEVs-GelMA hydrogel promotes scarless wound healing in aging rats

**Fig. 4.**
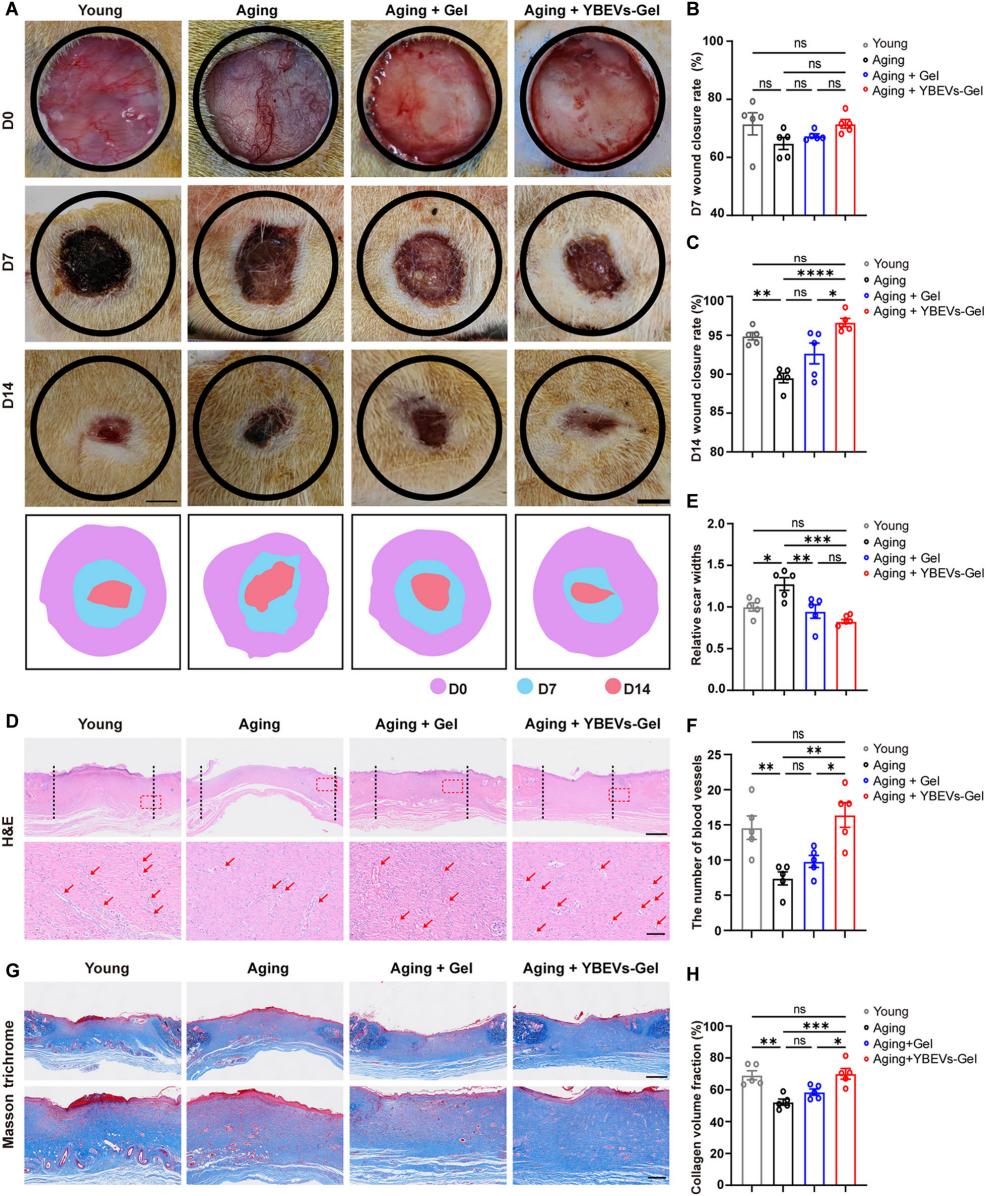
YBEVs promote wound healing in aging rats. (A) Representative wound closure images of different groups at each time point and corresponding wound area tracing. Scale bar, 5 mm. (B) Quantitative analysis of wound closure at day 7. (C) Quantitative analysis of wound closure at day 14. (D) H&E staining of repaired skin tissues at day 14, with the horizontal distance between the 2 vertical dotted lines indicating scar width. Red arrow, blood vessel. Scale bars, 1 mm and 100 μm. (E) Quantitative analysis of relative scar widths. (F) Quantitative analysis of vessels. (G) Masson trichrome staining on day 14 of injury. Scale bars, 1 mm and 400 μm. (H) Quantitative analysis of collagen volume fraction. All data are presented as means ± SEM; *n* = 5. *P < 0.05; **P < 0.01; ***P < 0.001; ****P < 0.0001; ns, *P* > 0.05. Young, the youthful group receiving no treatment; Aging, the aging group receiving no treatment; Aging + Gel, the aging group receiving GelMA hydrogel alone; Aging + YBEVs-Gel, the aging group receiving YBEVs-GelMA hydrogel.

A complete skin defect model in aged rats was used to assess the efficacy of YBEVs in vivo. For the experiment, we created 4 groups: the youthful group, which received no treatment (youthful); the aging group that received no treatment (Aging); the aging group that received GelMA hydrogel alone (Aging + Gel); and the aging group that received YBEVs-GelMA hydrogel (Aging + YBEVs-Gel). Dorsal skin wounds were photographed in each group of rats on days 0, 7, and 14 of injury (Fig. 4A). At day 7, all the wounds had crusted and contracted, as seen in Fig. 4B, with no discernible differences between the groups. However, compared to the Aging group, the Aging + YBEVs-Gel group showed significantly enhanced wound healing, as evidenced by reduced wound width (Fig. S3A) (*P* < 0.05). In contrast, there were no differences between the Aging + Gel group and the Aging group (*P* > 0.05), indicating that YBEVs-Gel exhibited superior wound repair capabilities compared to the Gel group alone. All wounds had essentially healed by day 14. The Aging group’s data showed a worse repair effect than that of the Young group, and the data from the Aging + Gel group and Aging group did not differ (about 90% and 88% wound closure rate, respectively). However, the Aging + YBEVs-Gel group had a significant advantage in terms of wound contraction, and they were able to reverse the aging skin’s poor repair effect and achieve the same level of repair as the Young group (approximately 95% wound closure rate) (Fig. 4C).

The above results suggest that YBEVs have a stronger ability to promote aging skin healing, while GelMA hydrogel does not have a similar ability. The Aging + YBEVs-Gel group significantly enhanced wound healing, achieving a wound width similar to that of the Young group. Moreover, hematoxylin and eosin (H&E) staining revealed that YBEVs significantly increased the number of blood vessels by day 14 post-injury (Fig. 4D to F). Notably, in the Aging group, the number and structure of hair follicles were reduced and impaired compared to the Young group. However, treatment with YBEVs restored the visibility of glandular structures resembling hair follicles and significantly increased the number of hair follicles (Fig. S4).

Collagen appears blue-green in tissue stained with Masson trichrome [34]. On the seventh day of injury, collagen fibers were heavily aggregated in the enlarged areas of the Young group and the Aging + YBEVs-Gel group, but these fibers did not form an ordered structure (Fig. S3B). At 14 d of injury, fibers in the Aging + YBEVs-Gel group had a more mature phenotype with a complex and well-organized collagen network structure compared with the other groups (Fig. 4G). Quantitative assessment of the collagen volume fraction supported these findings (Fig. 4H), suggesting that YBEVs enhanced collagen deposition. The deposition and orderly arrangement of collagen fibers can guide the proliferation and migration of epithelial cells and vascular endothelial cells, which can help wound epithelialization and reconstruction of vascular networks. The above results show that although GelMA hydrogel can promote skin healing in aging rats, its repairing effect is not as good as that of the GelMA hydrogel equipped with YBEVs, which can achieve the same healing effect as that of young rats.

**Fig. 5.**
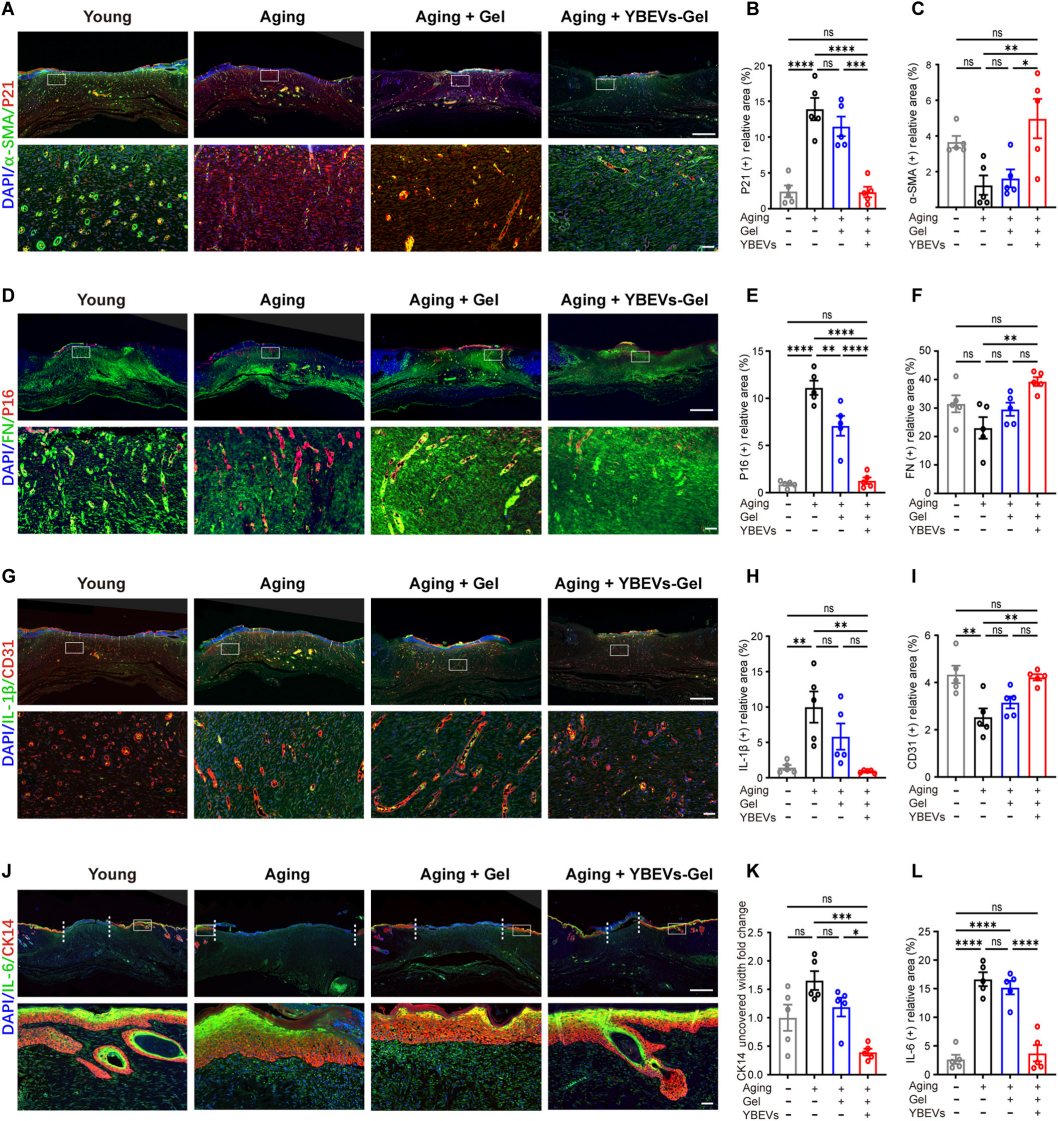
Immunofluorescence staining of in vivo experiments. (A) Representative images of P21 (red) and α-SMA (green) in the wound sections. Scale bars, 1 mm and 50 μm. (B) Quantitative analysis of P21 (+) relative area. (C) Quantitative analysis of α-SMA (+) relative area. (D) Representative images of P16 (red) and FN (green) in the wound sections. Scale bars, 1 mm and 50 μm. (E) Quantitative analysis of P16 (+) relative area. (F) Quantitative analysis of FN (+) relative area. (G) Representative images of CD31 (red) and IL-1β (green) in the wound sections. Scale bars, 1 mm and 50 μm. (H) Quantitative analysis of IL-1β (+) relative area. (I) Quantitative analysis of CD31 (+) relative area. (J) Representative images of CK14 (red) and IL6 (green) in the wound sections. Scale bars, 1 mm and 50 μm. (K) Quantitative analysis of CK14 uncovered width fold change. (L) Quantitative analysis of IL-6 (+) relative area. All data are presented as means ± SEM; *n* = 5. *P < 0.05; **P < 0.01; ***P < 0.001; ****P < 0.0001; ns, *P* > 0.05. Young, the youthful group receiving no treatment; Aging, the aging group receiving no treatment; Aging + Gel, the aging group receiving GelMA hydrogel alone; Aging + YBEVs-Gel, the aging group receiving YBEVs-GelMA hydrogel.

A series of fluorescence costaining experiments were performed to further explore the anti-aging, anti-inflammatory, vascularization, epidermal and dermal formation, and hair follicle formation functions of YBEVs. High expression of P21 and P16 is a major feature of SnCs [35], and it is usually accompanied by the expression of inflammation-related markers and increased senescence-associated secretory phenotype (SASP) [interleukin-1β (IL-1β) and IL-6] [36]. CD31 and α-smooth muscle actin (α-SMA) are classical indicators associated with neovascularization [37]. Cytokeratin 14 (CK14) is predominantly expressed in keratinocytes of the hair follicle matrix and the basal layer of the complex squamous epithelium [38], whereas FN is a marker associated with dermal formation [39]. YBEVs exhibited the lowest senescence-associated P21 and the highest angiogenesis-associated α-SMA expression, which was basically consistent with that of the Young group (Fig. 5A to C). The trends for P16 and FN were similar to those for P21 and α-SMA, respectively, further suggesting stronger anti-aging effects and pro-collagen formation of YBEVs to promote dermal repair (Fig. 5D to F).

Furthermore, YBEVs significantly inhibited SASP (IL-1β and IL-6) expression to the same level as the Young group (Fig. 5H and L), which means that YBEVs not only restored the SnCs themselves but also limited the negative effects that SnCs produce on the surrounding tissues and cells, further inhibiting the aging process. This feature of YBEVs is expected to be applied in the clinic to alleviate the rate of wound infections in the elderly and prolong their lifespans. YBEVs have also been shown to promote CD31 expression, but the morphology and quantification represented by CD31 was not dominant in the Aging + YBEVs-Gel group compared to the Young group (Fig. 5I). CK14 exhibited a clear distribution of specific areas in immunofluorescence staining, mainly concentrated on the wound surface. Its expression layer began to appear at the edge of the wound and then extended toward the center of the wound in an inner-thin and outer-thick distribution, showing a typical epithelial tongue (Fig. 5J). It was evident that CK14 crawled fastest in the Aging + YBEVs-Gel group, and its width was the smallest (Fig. 5K). A hallmark of skin wound scarring is the lack of functional appendages such as hair follicles and glands. The presence of hair follicle or sebaceous gland-like structures in the repair area in the Aging + YBEVs-Gel group and the expression of CK14 indicated the functional regeneration of hair follicles or sebaceous glands. This suggests that YBEVs not only promote rapid healing of senescent wounds but also favor scarless wound healing and repair. In summary, the fluorogram and its quantification showed that YBEVs inhibited senescence as well as inflammation-related protein expression in the skin repair area; promoted epidermal and dermal growth, neovascularization, and hair follicle formation; and ultimately led to rapid healing without scarring.

### YBEVs promote aged wound repair by alleviating mitochondrial dysfunction

In both in vivo and in vitro experiments, we saw that multiple aging-related and fiber-related indicators were significantly regulated by YBEVs. Therefore, we hypothesized that the effect of YBEVs on fiber cells is the result of a combination of actions that may alter the biological state or energy metabolism of fiber cells without focusing on a single gene. Subsequently, we focused on the protein cargo delivered by YBEVs when exploring the mechanism and the alterations associated with the overall biological state or energy metabolism of the cell.

**Fig. 6.**
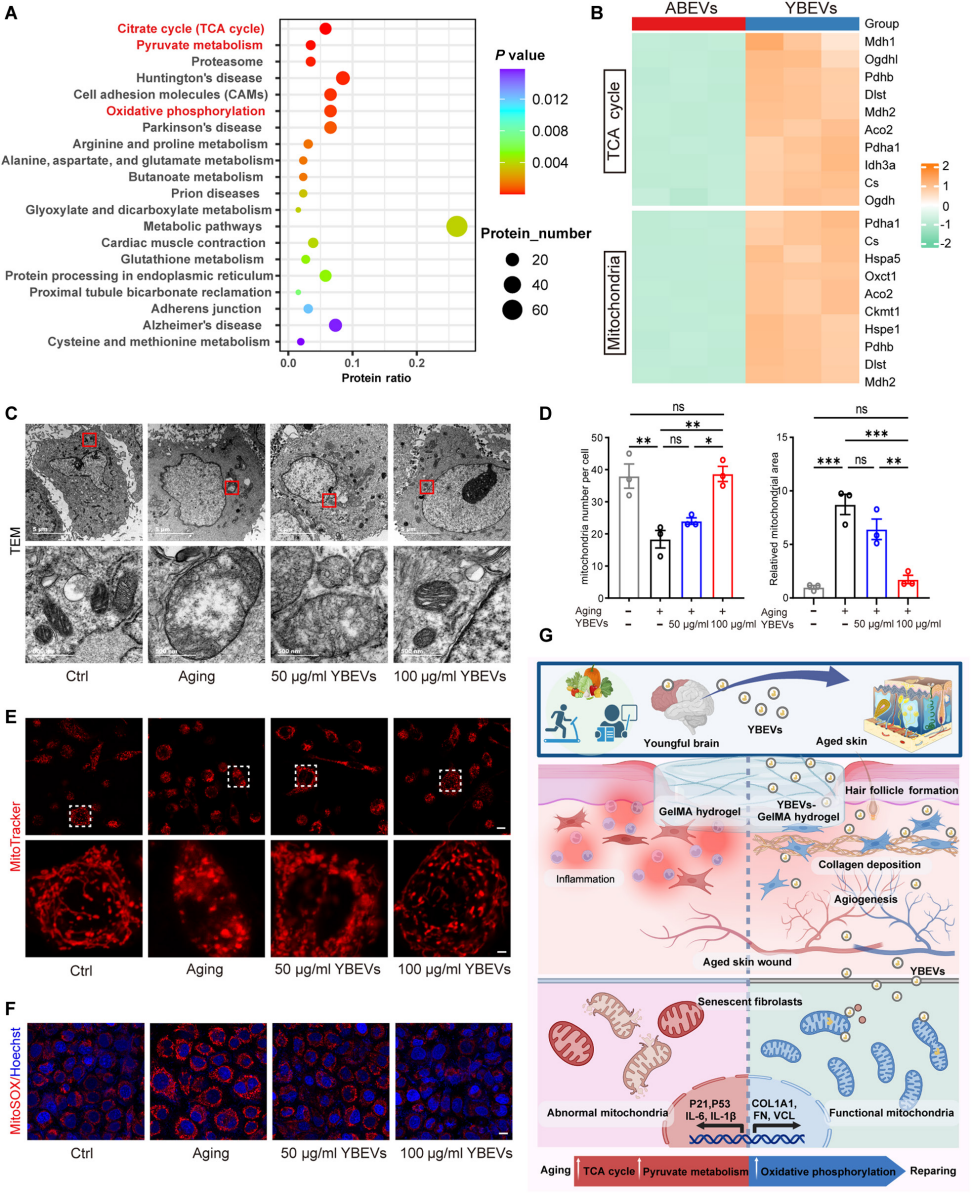
YBEVs ameliorate cell senescence via mitochondrial remodeling. (A) Identification of the top 20 KEGG pathways in the proteomic datasets for ABEVs and YBEVs. (B) Heatmap illustrating the enrichment of specific proteins. (C) Representative TEM images of mitochondria in HDFs treated with different stimuli. Scale bars, 5 μm and 500 nm. (D) Quantitative analysis of mitochondria number and mitochondria area. (E) MitoTracker images of treated HDFs. Scale bars, 10 μm and 2 μm. (F) MitoSOX images of treated HDFs. Scale bar, 10 μm. (G) Schematic diagram illustrating the anti-aging mechanism of YBEVs. All data are presented as means ± SEM; *n* = 3. *P < 0.05; **P < 0.01; ***P < 0.001. The schematic diagram illustration was created by BioRender.

For the protein array assay, Kyoto Encyclopedia of Genes and Genomes (KEGG) analysis showed that pathways enriched from different expression genes were clustered in certain processes, such as the tricarboxylic acid cycle (TCA cycle or citrate cycle), pyruvate metabolism, and oxidative phosphorylation (Fig. 6A), which means that there are substantial differences in protein-related mitochondrial function between YBEVs and aged brain-derived extracellular vesicles (ABEVs). Moreover, we found that the expression of proteins related to the citrate cycle, such as Mdh1 and mitochondria, and including Pdha1, was up-regulated in YBEVs compared with the ABEV group in the heatmap. The distribution of samples also suggests that the expression of the above genes is well homogenized and that the samples bring little variance (Fig. 6B). The results of the volcano plots were consistent with the heatmap, and the volcano plot showed that the expression profiles of these proteins varied considerably across groups (Fig. S5).

Overall, the results indicated that YBEVs contained a large number of mitochondria-related proteins, suggesting that YBEV treatment might enhance mitochondrial function in the wound area of aging skin. Mitochondria play an essential role in regulating metabolic processes, and mitochondrial dysfunction disrupts oxygen species metabolism, induces DNA damage, and accelerates the aging process [18]. During skin aging, mitochondrial dysfunction, characterized by mitochondrial DNA (mtDNA) damage and oxidative stress, impairs collagen and FN production while increasing proinflammatory cytokines, leading to reduced wound-healing ability in aging HDFs [40]. To investigate the effect of YBEVs on cellular senescence-induced mitochondrial dysfunction, mitochondrial morphology, reactive oxygen species (ROS) levels, membrane potential, and adenosine triphosphate (ATP) production were analyzed. TEM images and MitoTracker (red) staining revealed that the mitochondrial matrix of SnCs was swollen, increased in area, and exhibited irregular morphology, with disorganized or missing cristae. Treatment with 50 and 100 μg/ml YBEVs significantly improved mitochondrial damage, with the higher concentration showing a stronger effect (Fig. 6C and E). Additionally, YBEVs increased the number of mitochondria in cells and restored mitochondrial area to normal size (Fig. 6D). Furthermore, mitochondrial ROS (mtROS) staining indicated that senescence significantly increased mtROS levels, while YBEV treatment reduced this increase, with 100 μg/ml demonstrating a more pronounced effect than 50 μg/ml (Fig. 6F and Fig. S6B). JC-1 staining and ATP assays revealed that senescence significantly decreased mitochondrial membrane potential and ATP levels, indicating impaired mitochondrial energy function. While 50 μg/ml YBEVs did not rescue mitochondrial function (*P* > 0.05), 100 μg/ml YBEVs significantly increased membrane potential and ATP levels (*P* < 0.05) (Fig. S6A and C).

In conclusion, YBEVs ameliorate mitochondrial abnormalities in SnCs and promote tissue repair by delivering mitochondria-associated proteins and enhancing oxidative phosphorylation, thereby alleviating the senescent phenotype and inflammatory status (Fig. 6G). Our research provides new theoretical and experimental evidence to refine the “brain–skin axis” theory. However, this study has some limitations. The skin, as a neuroendocrine system, acts by preserving and maintaining the structural and functional integrity of the skin and, by inference, systemic homeostasis [7–9]. During wound healing, the skin might achieve different homeostatic states through autocrine mechanisms. Additionally, given the close relationship between the brain and the skin, and considering the skin as an endocrine organ, it is plausible that the hormones and other contents secreted by the skin after injury may feedback to the brain, potentially altering the cargoes in brain-EVs. However, the brain-EVs collected in this study are exogenous and derived from animals without injury induction. This issue remains to be further explored in future research.

## Conclusion

In conclusion, in our efforts to explore possible modulators and therapeutic strategies for impaired healing in aging skin and based on the concept of the “brain–skin axis”, we found that YBEVs, which contain a large number of TCA cycle- and mitochondria-related proteins, have a significant reduction effect on aging skin healing disorders, and the modulatory effect of YBEVs was immediate at small doses without toxic side effects at high doses. In addition, YBEVs-GelMA hydrogel was prepared, and this composite hydrogel could maintain the bioactivity of YBEVs and release them continuously at the wound site. Thus, our study explored not only the fundamental role of YBEVs as biomolecules but also their therapeutic potential, specifically highlighting the powerful improvement effects of maintaining a youthful brain state on aging skin. Therefore, it is reasonable to conclude that such YBEVs with anti-aging properties may pave the way for establishing therapeutic approaches for wound repair and regeneration in the elderly.

## Materials and Methods

### Cell culture and treatment

HDFs (ATCC PCS-201-012 ) were obtained from the American Type Culture Collection (ATCC) (Manassas, VA, USA) and maintained in Dulbecco’s modified Eagle’s medium (DMEM; Gibco, USA), supplemented with 10% fetal bovine serum (FBS; Gibco, USA) and 1% penicillin–streptomycin solution (Sigma, USA) at 37 °C in a humidified incubator with 5% CO_2_. The medium was refreshed every 2 d. To induce cellular senescence, HDFs were treated with 1 mM hydrogen peroxide (H_2_O_2_; Sigma-Aldrich, H1009) for 2 h.

### Isolation and characterization of YBEVs

YBEVs were isolated from the brains of 4-week-old Sprague–Dawley (SD) rats, obtained from Shanghai SLAC Laboratory Animal Corporation (Shanghai, China). The brains were dissected into 1- to 2-mm fragments and incubated in DMEM/F12 medium (Gibco, USA) containing 2 mg/ml papain (Sangon Biotech, Shanghai, China) at 37 °C for 30 min. Following dissociation, the tissue suspension was filtered through a 40-μm membrane (Millipore, USA) to remove larger debris. The filtrate was centrifuged sequentially at 300*g* for 10 min, 2,000*g* for 10 min, and 10,000*g* for 30 min to eliminate cells and residual debris. The supernatant was subjected to further centrifugation until no sediment remained. For final isolation, the supernatant was ultracentrifuged at 100,000*g* for 70 min (Beckman, USA), and the pelleted YBEVs were resuspended in phosphate-buffered saline (PBS). All centrifugation steps were performed at 4 °C, and YBEVs were used immediately after isolation. The concentration of YBEVs was determined using a BCA protein assay kit (Beyotime, China). To characterize the YBEVs, nanoparticle tracking analysis (NTA; Malvern NanoSight NS500, UK) was employed to assess particle size and distribution. Additionally, TEM (Tecnai G2 Spirit 120 kV, Thermo Fisher Scientific, USA) was used to examine the morphology of the EVs. Surface marker expression on YBEVs was assessed by WB, using antibodies against annexin V (Cell Signaling Technology #8555, USA), flotillin-1 (Cell Signaling Technology #3253, USA), GM130 (Cell Signaling Technology #70767, USA), and EpCAM (Cell Signaling Technology #93790, USA).

### Cellular uptake of YBEVs by HDFs

YBEVs were labeled with PKH26 (10 mM; Sigma-Aldrich, USA) according to the manufacturer’s instructions. Labeled EVs were incubated with HDFs at 37 °C for 8 h. Subsequently, the cells were washed with PBS and fixed in 4% paraformaldehyde (Solarbio, China) for 15 min. The cytoskeleton was stained with phalloidin (Invitrogen, USA) for 20 min, followed by 3 PBS washes, and the nucleus was counterstained with 4′,6-diamidino-2-phenylindole (DAPI) (Solarbio, China) for 5 min. After PBS washing and anti-quenching treatment, the endocytosis of target cells was observed using a 63× oil-immersion objective on a confocal microscope (Thermo Fisher Scientific, USA).

### SA-β-Gal staining

SA-β-Gal activity was assessed using a β-galactosidase staining kit (Beyotime, China) following the manufacturer’s protocol. Cells were washed with PBS, fixed with fixation solution for 15 min at 37 °C, and incubated overnight at 37 °C in staining solution. Blue-stained cells were photographed and counted under a 20× magnification.

### Cell immunofluorescence staining

Cells from different experimental groups were fixed, permeabilized, blocked, and incubated overnight at 4 °C with primary antibodies against P21 (Abcam ab109520, USA) and FN (Abcam ab2413, USA). After incubation with secondary antibody for 1 h at room temperature, cells were treated with phalloidin (Proteintech, China) for 20 min and DAPI (Solarbio, China) for 5 min. Images were captured using a Keyence BZ-X800 All-in-One Fluorescence Microscope (Keyence, Japan), and fluorescence area quantification was performed using ImageJ software.

### Assessment of the proliferation ability

HDFs in the logarithmic growth phase were seeded in 96-well plates. After 24 h, cells were treated with 1 mM H_2_O_2_, with or without varying concentrations of YBEVs. Following an additional 2 h of culture, 10 μl of Cell Counting Kit-8 (CCK-8) reagent was added, and the plate was incubated at 37 °C for 1.5 h. Absorbance was measured at 450 nm using a microplate reader.

### Cell migration assay

HDFs were seeded in 6-well plates and cultured until confluence. A scratch was made using a pipette tip at the center of the well, and cells were treated with medium containing low serum and H_2_O_2_, with or without different concentrations of YBEVs. Images were taken daily, and the migration area was quantified using ImageJ software. The migration rate was calculated as follows: Migration rate (%) = (*A*_0_ − *A*_t_)/*A*_0_ × 100%, where *A*_0_ is the initial area and *A*_t_ is the remaining area at the time of measurement.

### RT-qPCR analysis

Total RNA was extracted from HDFs treated with 1 mM H_2_O_2_ in the presence or absence of YBEVs, using TRIzol reagent (Invitrogen, USA). Reverse transcription was performed using the PrimeScript RT Master Mix (TaKaRa Biotechnology, Japan). RT-qPCR was conducted with SYBR Green Ex Taq Premix to measure the mRNA expression levels of P21, P53, COL1A1, VCL, and FN, with glyceraldehyde-3-phosphate dehydrogenase (GAPDH) as an internal control. Primer sequences (Sangon Biotech Co. Ltd., China) are listed in Table S1.

### Western blotting

Total cell proteins were extracted using radioimmunoprecipitation assay (RIPA) buffer containing a 1% protease inhibitor cocktail. The protein concentrations of the samples were quantified using Micro BCA Protein Assay Kits (Thermo Fisher Scientific). The samples were then separated in a 4% to 15% gradient sodium dodecyl sulfate–polyacrylamide gel electrophoresis (SDS-PAGE) gel (Bio-Rad) and transferred to Hybond-P polyvinyl difluoride (PVDF) membranes (Bio-Rad). After blocking in 5% skimmed milk for 1 h, the membranes were incubated with rabbit anti-P21 (Proteintech, 10355-1-AP), rabbit anti-P53 (Proteintech, 10442-1-AP), and mouse anti-β-actin (HUABIO) antibodies at a 1:1,000 dilution overnight at 4 °C. The membranes were washed with tris-buffered saline–Tween 20 (TBST) 3 times and incubated with anti-rabbit/anti-mouse immunoglobulin–horseradish peroxidase for 1 h at room temperature. A Bio-Rad imaging system was used for target protein detection. Images were analyzed using ImageJ software.

### Mitochondrial functional imaging assays

To assess mtROS production, HDFs were incubated with 1 mM MitoSOX Red (GLPBIO, USA) for 30 min, followed by Hoechst staining for 5 min. Fluorescence signals of Hoechst and MitoSOX were captured under laser scanning confocal microscopy using excitation wavelengths of 405 and 510 nm, respectively. Mitochondrial membrane potential was evaluated by incubating cells with JC-1 probe (Beyotime, China) for 20 min, followed by removal of the probe. JC-1 monomers were detected at 490 nm (excitation) and 530 nm (emission), while aggregates were detected at 525 nm (excitation) and 590 nm (emission). Quantitative analysis of fluorescence intensity was performed using ImageJ software, and mitochondrial membrane potential was assessed by the red-to-green fluorescence ratio.

### ATP detection assay

Cellular ATP levels under different stimuli were assessed using an ATP Detection Kit (Beyotime, China). Briefly, 100 μl of ATP detection buffer and 20 μl of sample were added to each well according to the manufacturer’s protocol. Luminescence was subsequently measured, and ATP levels were quantified relative to a standard curve.

### Mitochondrial morphology imaging assays

To investigate mitochondrial morphology, HDFs were incubated with 250 nM MitoTracker (GenVivo Tech, China) at 37 °C for 20 min. Following incubation, mitochondrial morphology and distribution were examined using confocal microscopy with excitation at 637 nm.

### Transmission electron microscopy

Cells were harvested, pelleted, and fixed in 2.5% glutaraldehyde in PBS at 4 °C. The fixed pellets were dehydrated through a graded ethanol series (50%, 70%, 90%, and 100%) and embedded in resin for 48 h at 65 °C. Ultra-thin sections (70 to 90 nm thick) were cut using an Ultramicrotome (EM UC7, Leica, Germany) and stained with lead citrate and uranyl acetate solutions for 5 to 10 min, respectively. Mitochondrial morphology and counts in different experimental groups were analyzed using a Tecnai Spirit 120 kV TEM.

### Proteomic analysis of YBEVs

ABEVs and YBEVs were isolated by ultracentrifugation, followed by protein extraction, enzymatic desalting, and fractionation. Nanoflow liquid chromatography–tandem mass spectrometry (LC-MS/MS) analysis was performed on a QE HF-X mass spectrometer (Thermo Fisher Scientific, Bremen, Germany). Raw data were processed using DIA-NN software (v1.8.1). After normalization of raw data, differential proteins were screened, annotated, and subjected to functional enrichment analyses, including Gene Ontology (GO) and KEGG enrichment, protein–protein interaction (PPI) analysis, and subcellular localization analysis using LocTree3 (score > 40). KEGG pathway, GO enrichment plots, and heat and volcano plots of TCA cycle and mitochondria-associated proteins were generated. The proteomic analysis was conducted by Beijing Enze Kangtai Biotechnology Company.

### Synthesis of GelMA hydrogel and YBEVs-GelMA hydrogel

GelMA (Engineering for Life, China) was dissolved in double-distilled water (ddH_2_O) containing 0.25% (w/v) lithium phenyl-2,4,6-trimethylbenzoylphosphinate (LAP) photoinitiator at 60 °C for 20 min in the dark. The resulting GelMA hydrogel solution was then sterilized using a 0.22-μm membrane filter (Millipore, USA) and irradiated under UV light for 30 s. To prepare YBEVs-GelMA hydrogel, the GelMA hydrogel solution was mixed with 100 μg/ml YBEVs and stirred for 30 s before being stored at −20 °C after gelation.

### Characterization of GelMA hydrogel and YBEVs-GelMA hydrogel

GelMA hydrogel and YBEVs-GelMA hydrogel samples were lyophilized overnight, gold-coated, and analyzed using scanning electron microscopy (SEM; ZEISS Gemini 300, Germany). Elemental composition (C, N, O, P) of the hydrogels was determined by EDS. Fourier-transform infrared spectroscopy (FTIR; Nicolet iS50, Thermo, USA) was employed to examine the molecular structure and chemical composition of both GelMA hydrogel and YBEVs-GelMA hydrogel.

### Hydrogel degradation assay and release analysis

GelMA hydrogel and YBEVs-GelMA hydrogel were placed in 5 ml of PBS and incubated at 37 °C with shaking. At predetermined time points, samples were removed, freeze-dried, and weighed. The degradation ratio was calculated as follows: Degradation ratio = current weight/initial weight × 100%. To evaluate YBEV release from the hydrogel, 100 mg of YBEVs was mixed with 10% GelMA and immersed in 5 ml of PBS. The supernatant was collected daily for 14 d, and protein concentration was measured using a BCA Protein Assay Kit (Solarbio, China). YBEV release was quantified based on protein levels.

### Animal skin defect model

All animal experiments were approved by the Laboratory Animals Ethics Committee of Zhejiang University (approval no. ZJU20240880, Hangzhou, China) and conducted in accordance with the Chinese National Institutes of Health Guidelines for the Care and Use of Laboratory Animals. Healthy young (4 weeks) and aged (20 months) SD rats were used as models. Rats were randomly assigned to 4 groups and anesthetized by intraperitoneal injection of 10 g/l pentobarbital sodium (0.4 ml/100 g). Dorsal skin was shaved and disinfected with iodine, and a full-thickness wound (2 cm diameter) was excised symmetrically on both sides of the back. No treatment was applied to wounds in the young and aged control groups. GelMA hydrogel and YBEVs-GelMA hydrogel (1.5 ml) were applied to the wounds, respectively, followed by UV irradiation. The wounds were covered with cotton gauze, and rats were euthanized at days 7 and 14. Wound healing was assessed by photographing and quantifying the wound area using ImageJ software. Wound closure rate was calculated as follows: Wound closure rate (%) = (*A*_0_ − *A*_t_)/*A*_0_ × 100%, where *A*_0_ is the initial area and *A*_t_ is the remaining area at the measured time point.

### Histological assessment

Harvested tissues were fixed in 4% paraformaldehyde for 24 h, embedded in paraffin, and sectioned into 5-μm slices. Histopathological examination was performed using H&E (Sevier, China) and Masson trichrome staining (Sevier, China). Quantitative analysis of scar width, blood vessel density, and collagen deposition was performed using ImageJ software.

### Immunofluorescent staining

Tissue sections were permeabilized with 0.2% Triton X-100 in PBS and blocked with 10% horse serum (Gibco, USA). Sections were incubated overnight at 4 °C with primary antibodies against FN (Sevier GB114491-100, China), P16 (Proteintech 10883-1-AP, China), α-SMA (Sevier GB111364-100, China), P21 (Sevier GB155313-100, China), CD31 (Sevier GB120005-100, China), IL-1β (Proteintech 16806-1-AP, China), CK14 (Sevier GB15803-100, China), and IL-6 (Sevier GB11117-100, China). Nuclei were counterstained with DAPI (2 μg/ml, Sigma, USA) for 10 min, and sections were mounted with Antifade ProLong Reagent (Invitrogen, USA). Fluorescence images were captured using a VS200 slide scanner (Olympus, Japan). Quantification of fluorescence intensity and distance was performed using ImageJ software.

### Statistical analysis

Statistical analysis was performed using GraphPad Prism 9.5 software. Data are presented as mean ± SEM. Differences between groups were analyzed using Student’s *t* test or one-way analysis of variance (ANOVA), with a *P* value of <0.05 considered statistically significant.

## Data Availability

The data supporting the findings of this study are available from the corresponding author upon reasonable request.
