# Supplementary material for: Youthful Brain-Derived Extracellular Vesicle-Loaded GelMA Hydrogel Promotes Scarless Wound Healing in Aged Skin by Modulating Senescence and Mitochondrial Function
Source: Research (Wash D C). 2025 Mar 28;8:0644. doi: 10.34133/research.0644 (PMC11951976; doi:10.34133/research.0644)
Supplement: Supplementary 1 — Figs. S1 to S6 Table S1 [file research.0644.f1.zip › Supplementary Materials.docx]

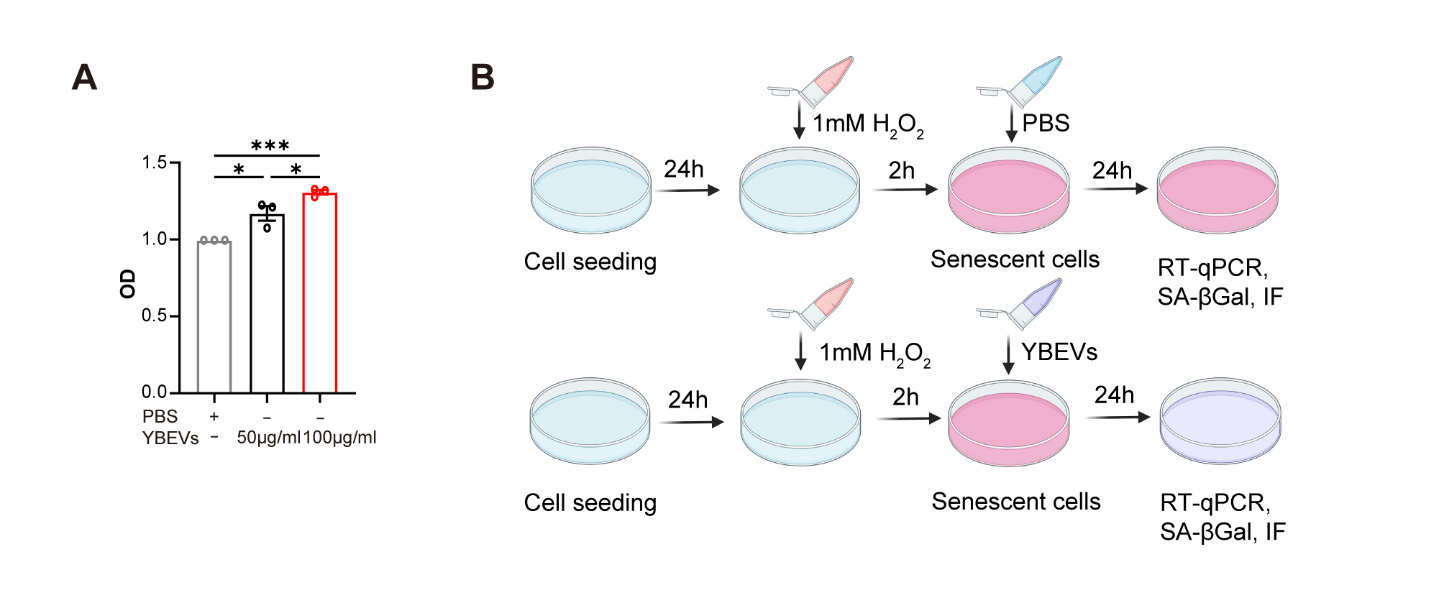


**Fig. S1.** Toxicological evaluation of YBEVs and schematic diagram of *in vitro* anti-aging experiments. (A)CCK8 was performed to analyze the cell viability of HDFs, which were cultured with varying concentrations of YBEVs (50 and 100 μg/mL). (B)Schematic diagram depicting the construction of an *in vitro* aging model and the anti-aging effects of YBEVs. All data are presented as means ± SEM; n = 3. **P < 0.05.***P* < 0.001. YBEVs, youthful brain-derived extracellular vesicles. The Created schematic diagram illustrates was created by BioRender.com.


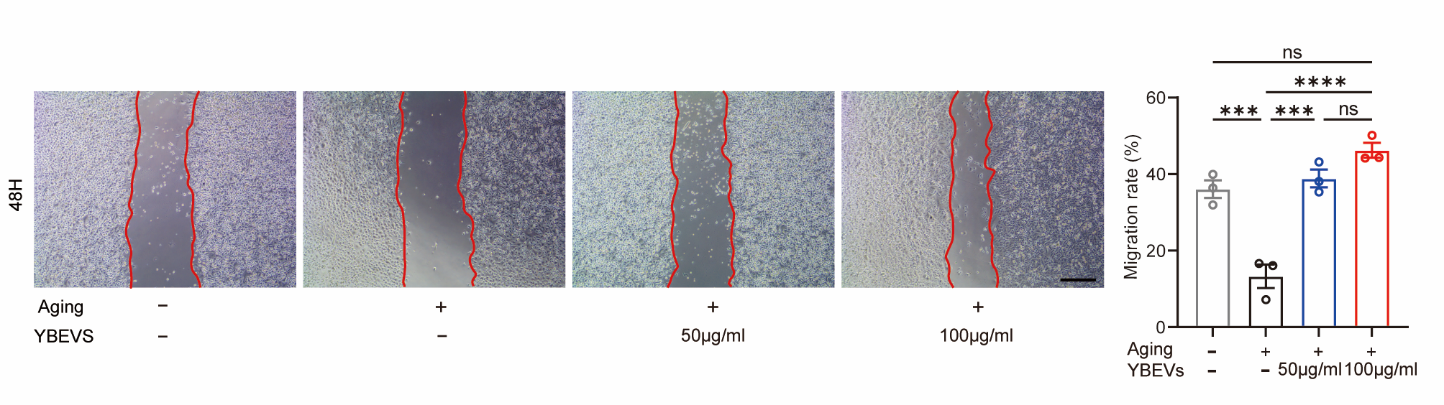


**Fig. S2.** Cell scratching assay and quantitative analysis (48 h). Scale bar: 200 μm. All data are presented as means ± SEM; n = 3. ****P* < 0.001. *****P* < 0.0001. *ns, P* > 0.05. YBEVs, youthful brain-derived extracellular vesicles.


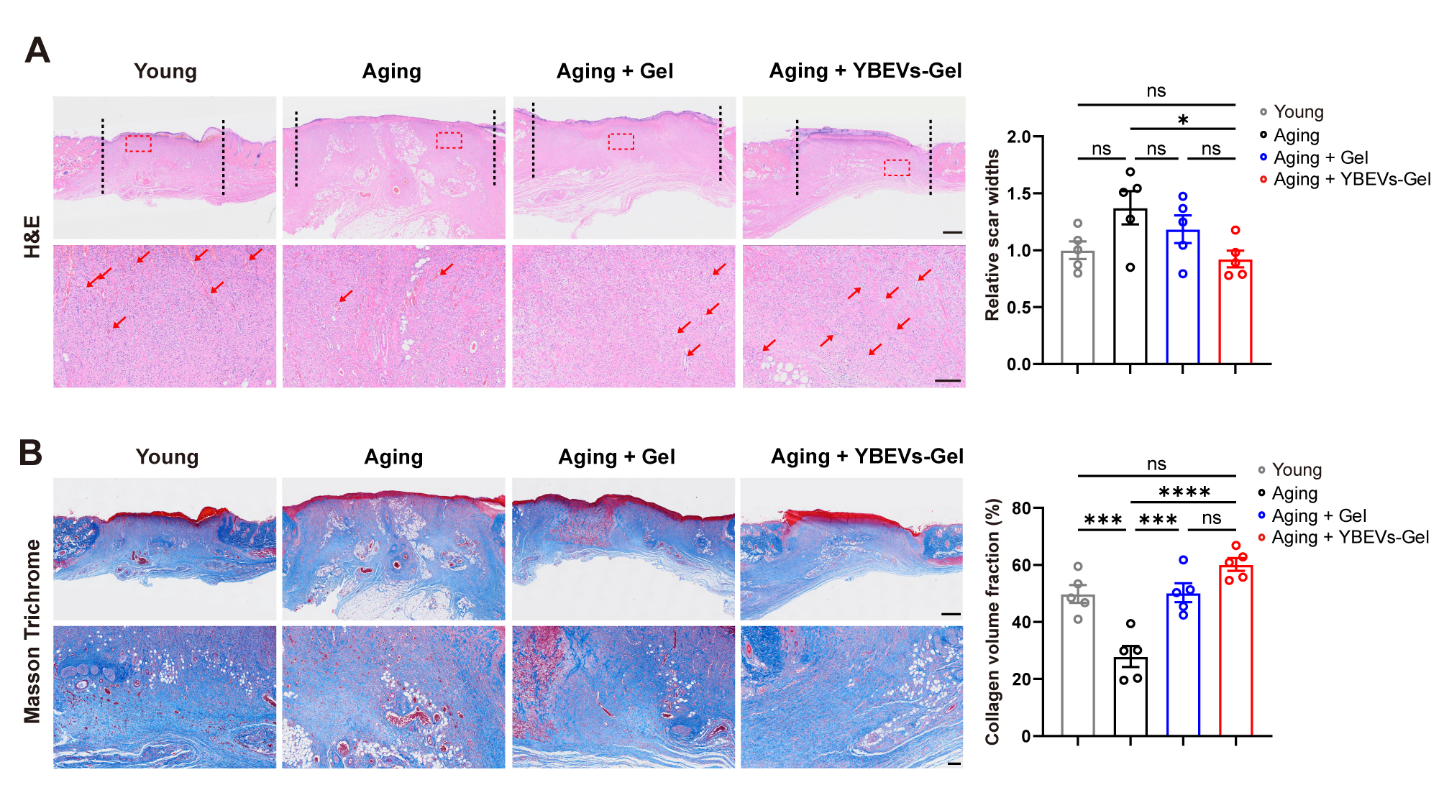


**Fig. S3.** HE and Masson trichrome staining on day 7 of injury. (A) HE representative images and relative scar width quantification. Scale bar: 1 mm and 200 μm. (B) Masson trichrome staining representative images and quantitative analysis of collagen volume fraction. Scale bar: 1 mm and 200 μm. All data are presented as means ± SEM; n = 5. **P* < 0.05. ****P* < 0.001. *****P* < 0.0001. *ns, P* > 0.05. Young: the youthful group receiving no treatment; Aging: the aging group receiving no treatment; Aging + Gel: the aging group receiving GelMA hydrogel alone; Aging + YBEVs-Gel: the aging group receiving YBEVs-GelMA hydrogel.

**
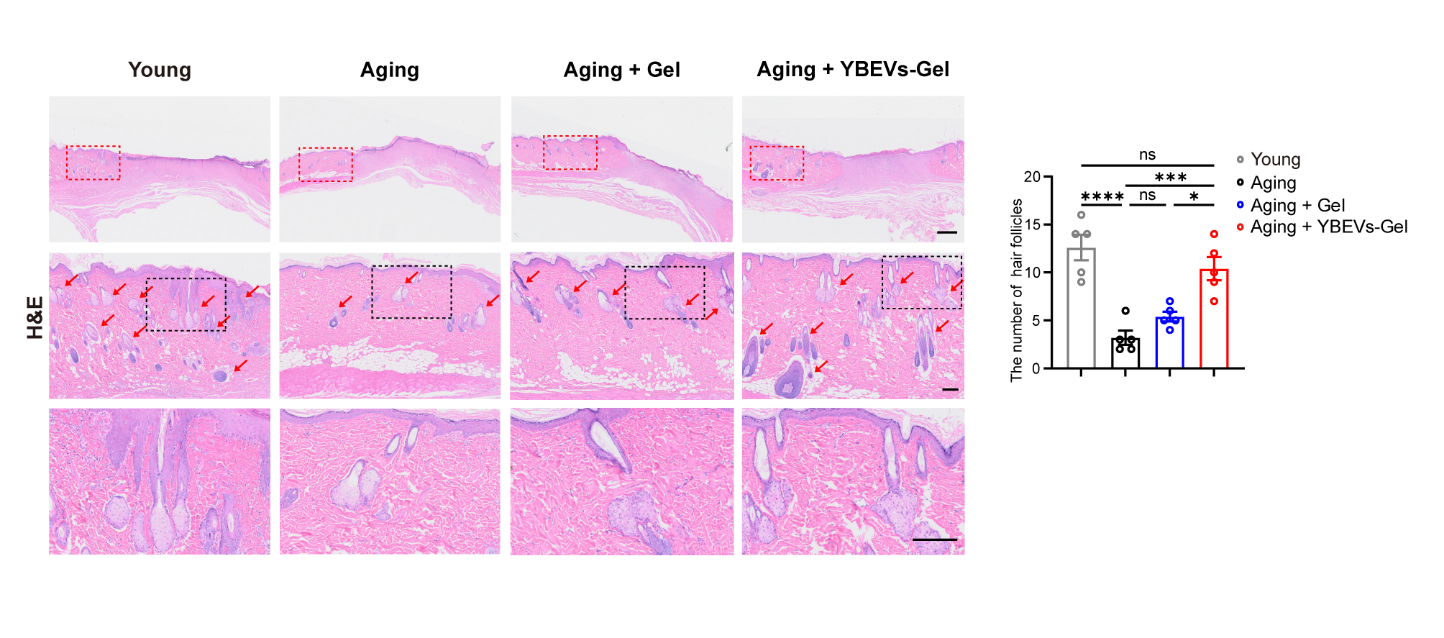
**

**Fig. S4.** Representative images and quantification of hair follicles. Scale bars: 1 mm and 200 μm. All data are presented as means ± SEM; n = 5. **P* < 0.05. ****P* < 0.001. *****P* < 0.0001. *ns, P* > 0.05. Young: the youthful group receiving no treatment; Aging: the aging group receiving no treatment; Aging + Gel: the aging group receiving GelMA hydrogel alone; Aging + YBEVs-Gel: the aging group receiving YBEVs-GelMA hydrogel.

**
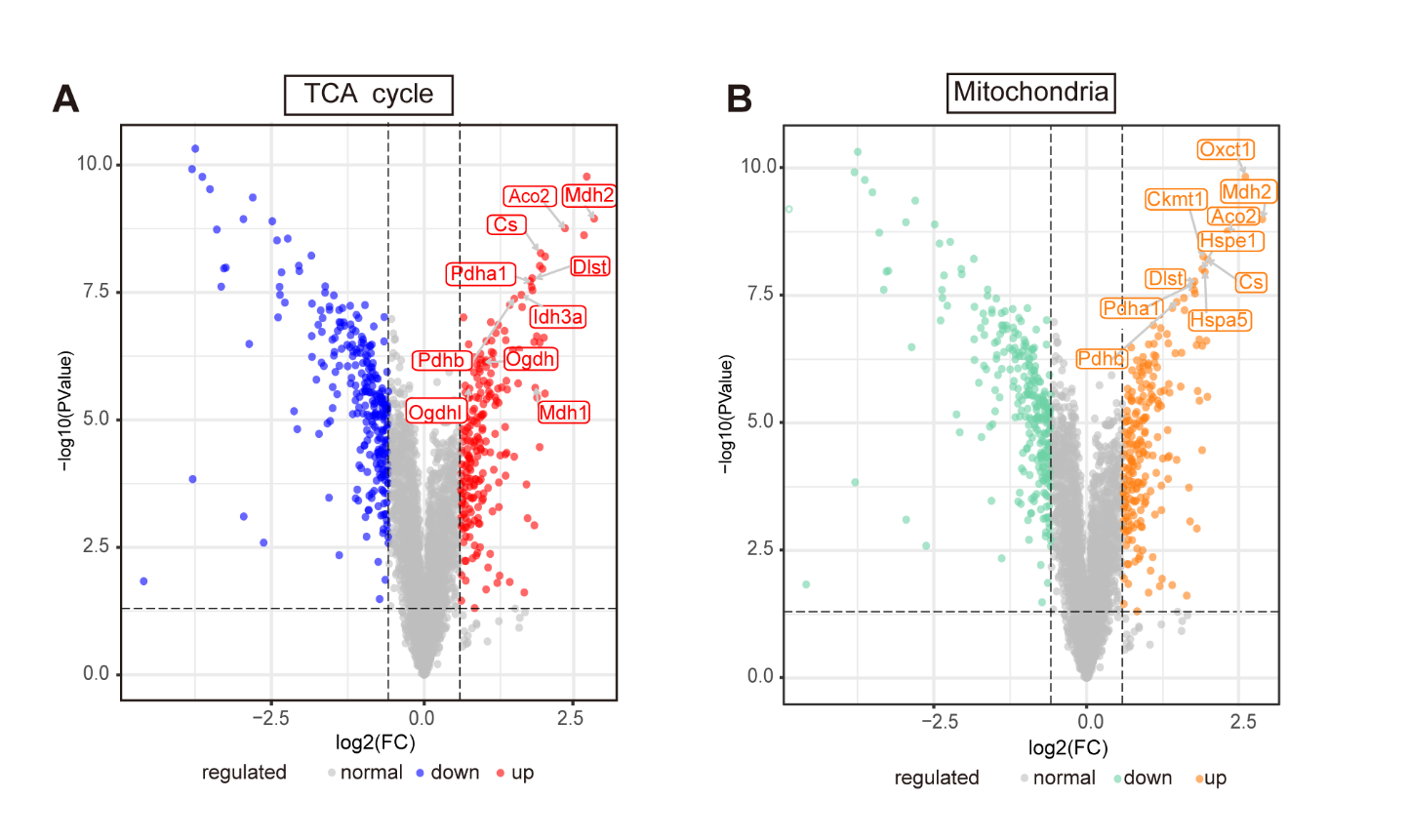
**

**Fig. S5.** Differential gene expression analysis between ABEVs and YBEVs. (A, B) Volcano plot analysis between ABEVs and YBEVs group in TCA cycle and mitochondria. ABEVs, aged brain-derived extracellular vesicles; YBEVs, youthful brain-derived extracellular vesicles.

**
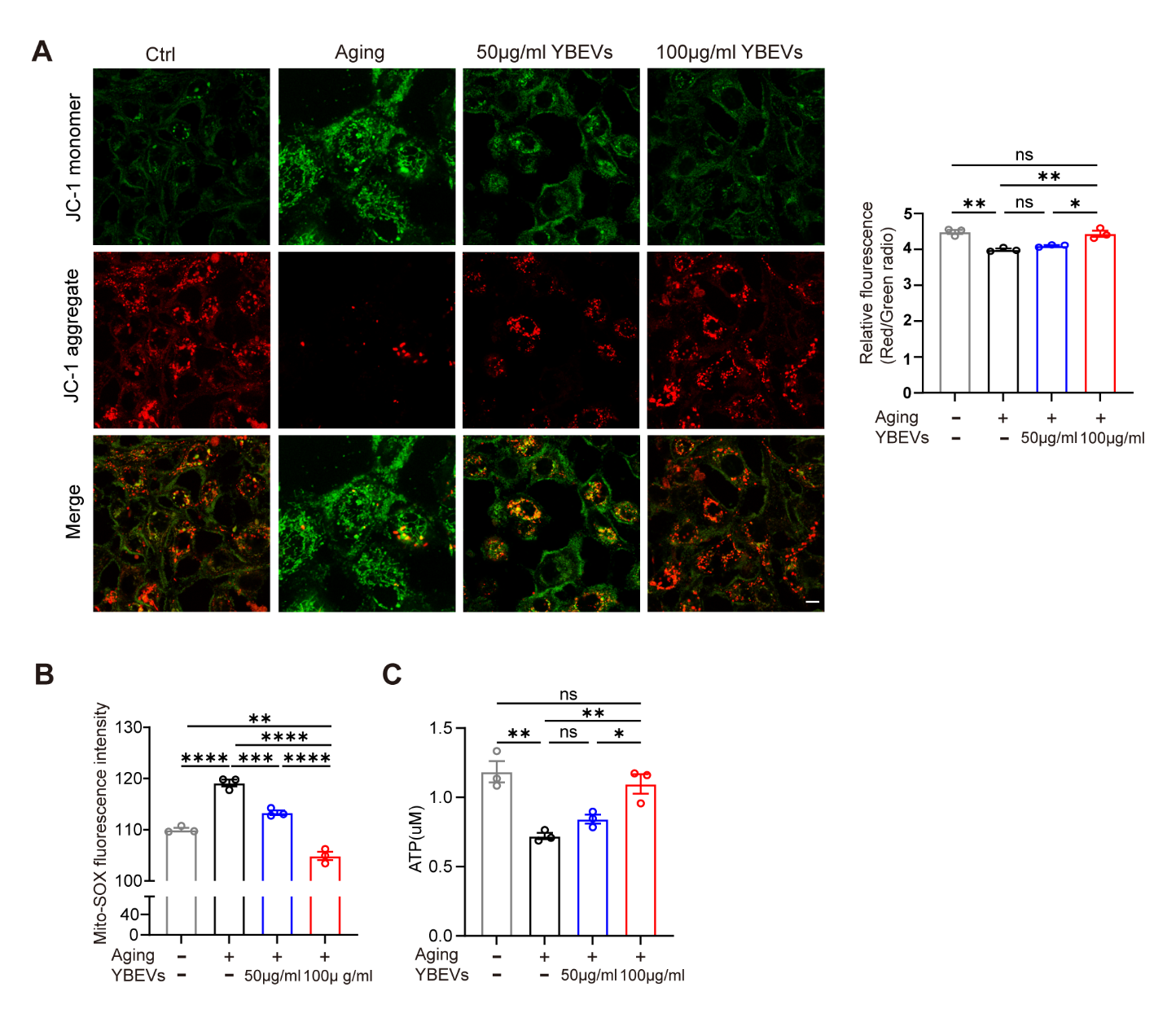
**

**Fig. S6.** YBEVs attenuate cell senescence through mitochondrial structural and functional reorganization. (A) JC-1 representative fluorescence images and quantitative analysis. Scale bar: 10 μm. (B) Quantification of MitoSOX fluorescence intensity. (C) Quantification of ATP levels. All data are presented as means ± SEM; n = 3. **P* < 0.05. ***P* < 0.01. ****P* < 0.001. YBEVs, youthful brain-derived extracellular vesicles.

**Table S1.** **Primers sequence used for qRT-PCR in this study**

| **Gene** | **Primers (5’-3’) (F=forward; R=reverse)** |
| --- | --- |
| **P53**  **COL1A1**  **VCL**  **FN1**  **GADPH** | F: CCTCAGCATCTTATCCGAGTG  R: TGGATGGTGGTACAGTCAGAGC  F: TGGCAAAGAAGGCGGCAAAGG  R: AGGAGCACCAGCAGGACCATC  F: GCTCTGCTGATGGCTGAGATGTC  R: GGCGATGTCCTTGGCACACTG  F: AGAGGCATAAGGTTCGGGAAGAGG  R: CGAGTCATCCGTAGGTTGGTTCAAG  F: GTCTCCTCTGACTTCAACAGCG  R: ACCACCCTGTTGCTGTAGCCAA |
|  |  |
