# Supplementary material for: Youthful Brain-Derived Extracellular Vesicle-Loaded GelMA Hydrogel Promotes Scarless Wound Healing in Aged Skin by Modulating Senescence and Mitochondrial Function
Source: Research (Wash D C). 2025 Mar 28;8:0644. doi: 10.34133/research.0644 (PMC11951976; doi:10.34133/research.0644)
Supplement: Supplementary 1 — Figs. S1 to S6 Table S1 [file research.0644.f1.zip › Table SI.docx]

**Table S1. Primers sequence used for qRT-PCR in this study**

| **Gene** | **Primers (5’-3’) (F=forward; R=reverse)** |
| --- | --- |
| ***P53***  ***COL1A1***  ***VCL***  ***FN1***  ***GADPH*** | F: CCTCAGCATCTTATCCGAGTG  R: TGGATGGTGGTACAGTCAGAGC  F: TGGCAAAGAAGGCGGCAAAGG  R: AGGAGCACCAGCAGGACCATC  F: GCTCTGCTGATGGCTGAGATGTC  R: GGCGATGTCCTTGGCACACTG  F: AGAGGCATAAGGTTCGGGAAGAGG  R: CGAGTCATCCGTAGGTTGGTTCAAG  F: GTCTCCTCTGACTTCAACAGCG |
|  | R: ACCACCCTGTTGCTGTAGCCAA |
