# Supplementary figures and images for: Youthful Brain-Derived Extracellular Vesicle-Loaded GelMA Hydrogel Promotes Scarless Wound Healing in Aged Skin by Modulating Senescence and Mitochondrial Function
Source: Research (Wash D C). 2025 Mar 28;8:0644. doi: 10.34133/research.0644 (PMC11951976; doi:10.34133/research.0644)

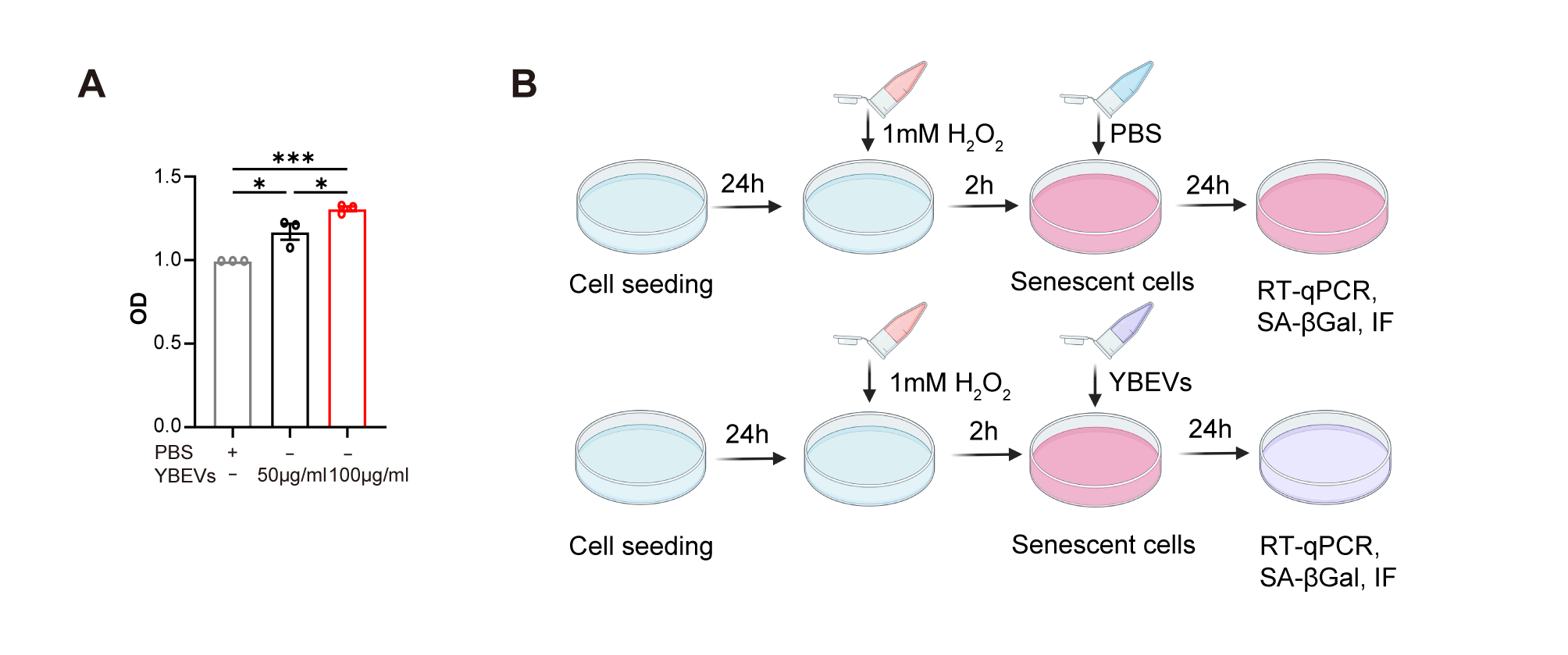

Supplement: Supplementary 1 — Figs. S1 to S6 Table S1 [file research.0644.f1.zip › S1.tif]

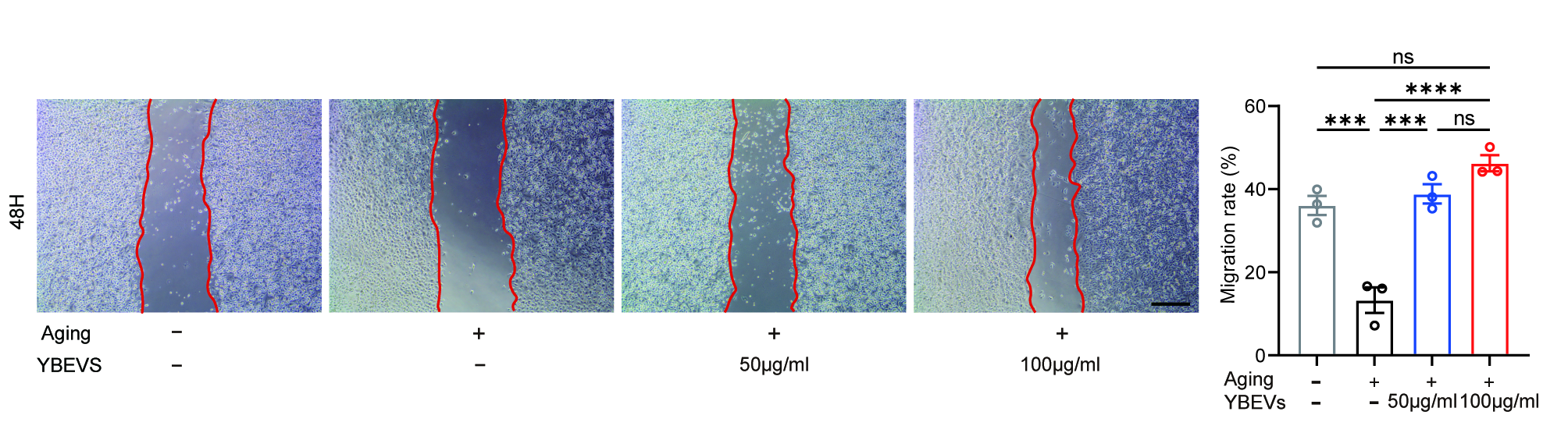

Supplement: Supplementary 1 — Figs. S1 to S6 Table S1 [file research.0644.f1.zip › S2.tif]

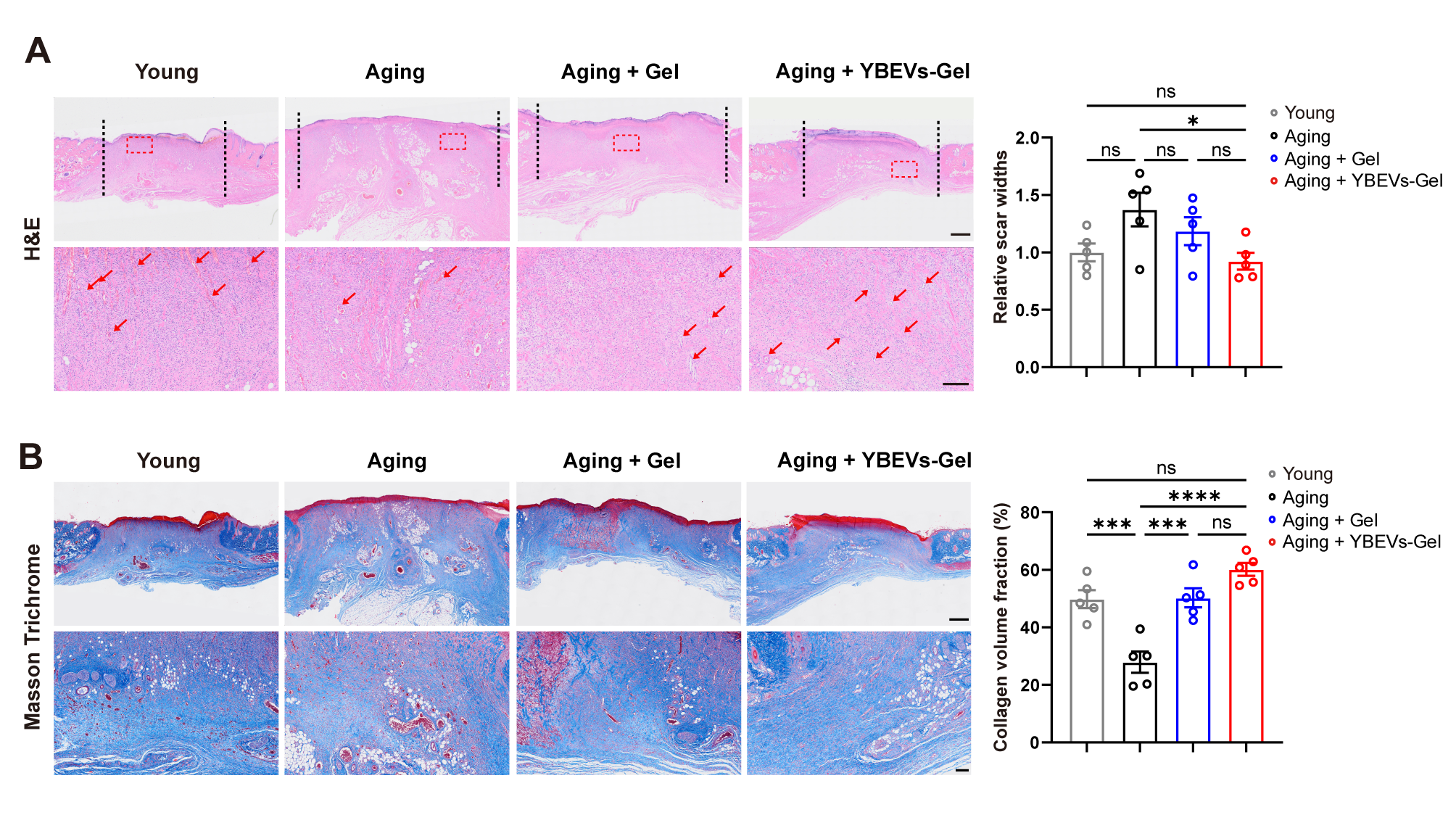

Supplement: Supplementary 1 — Figs. S1 to S6 Table S1 [file research.0644.f1.zip › S3.tif]

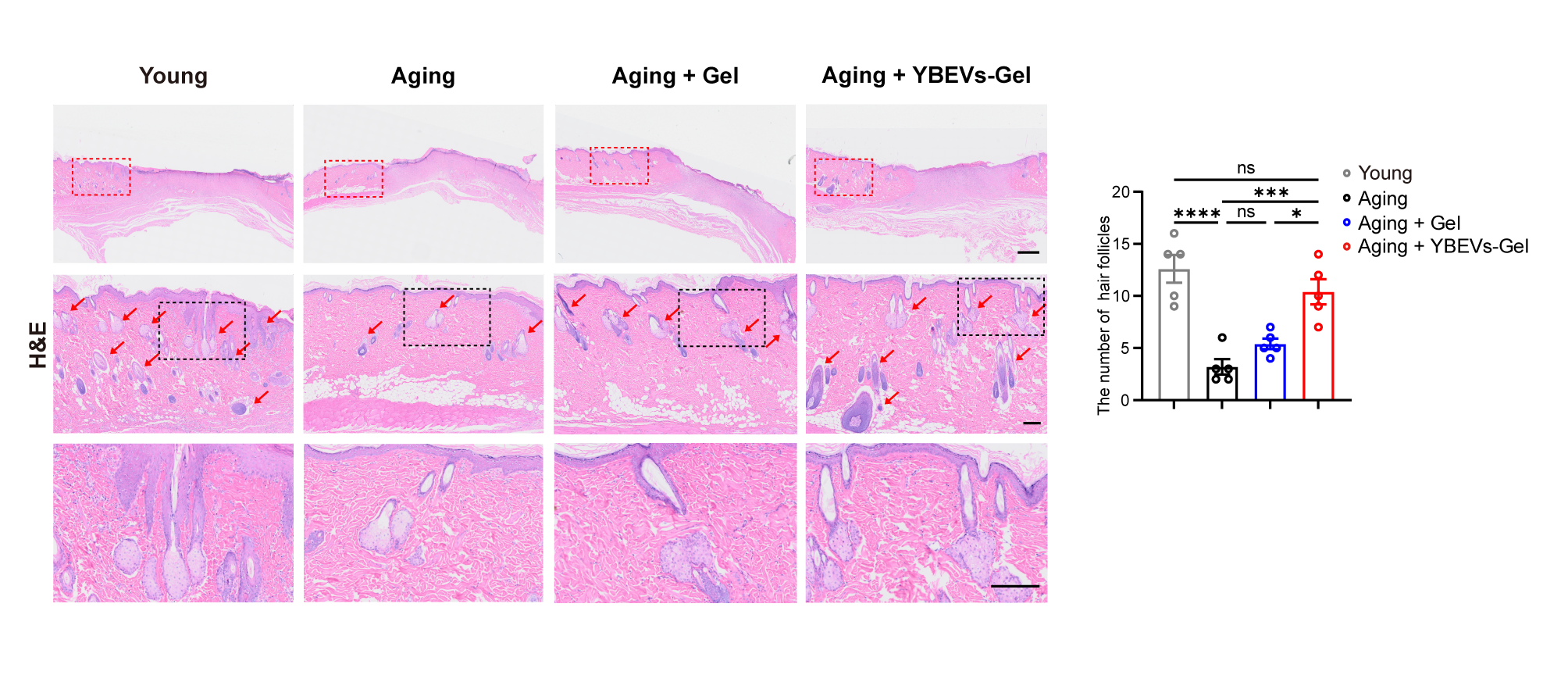

Supplement: Supplementary 1 — Figs. S1 to S6 Table S1 [file research.0644.f1.zip › S4.tif]

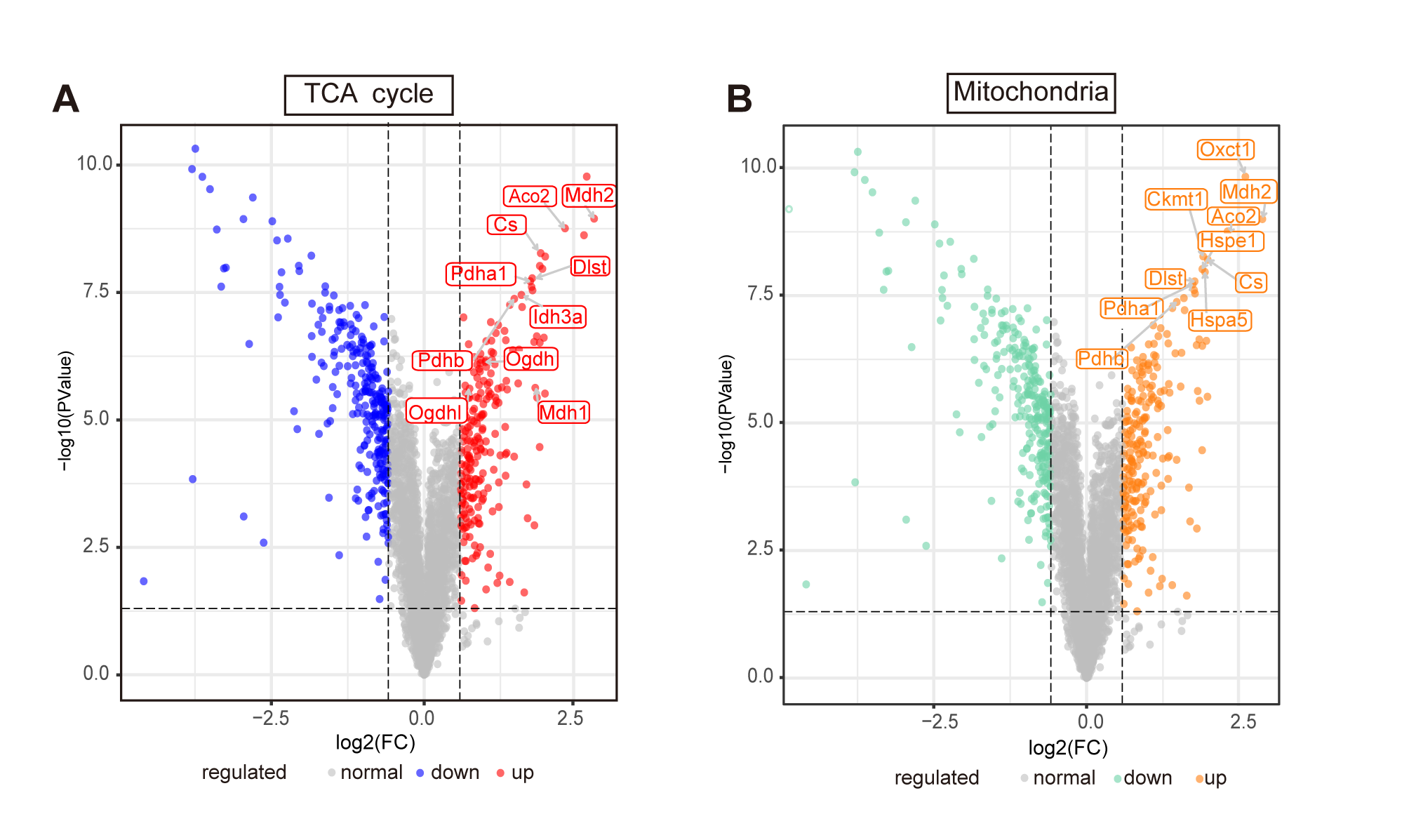

Supplement: Supplementary 1 — Figs. S1 to S6 Table S1 [file research.0644.f1.zip › S5.tif]

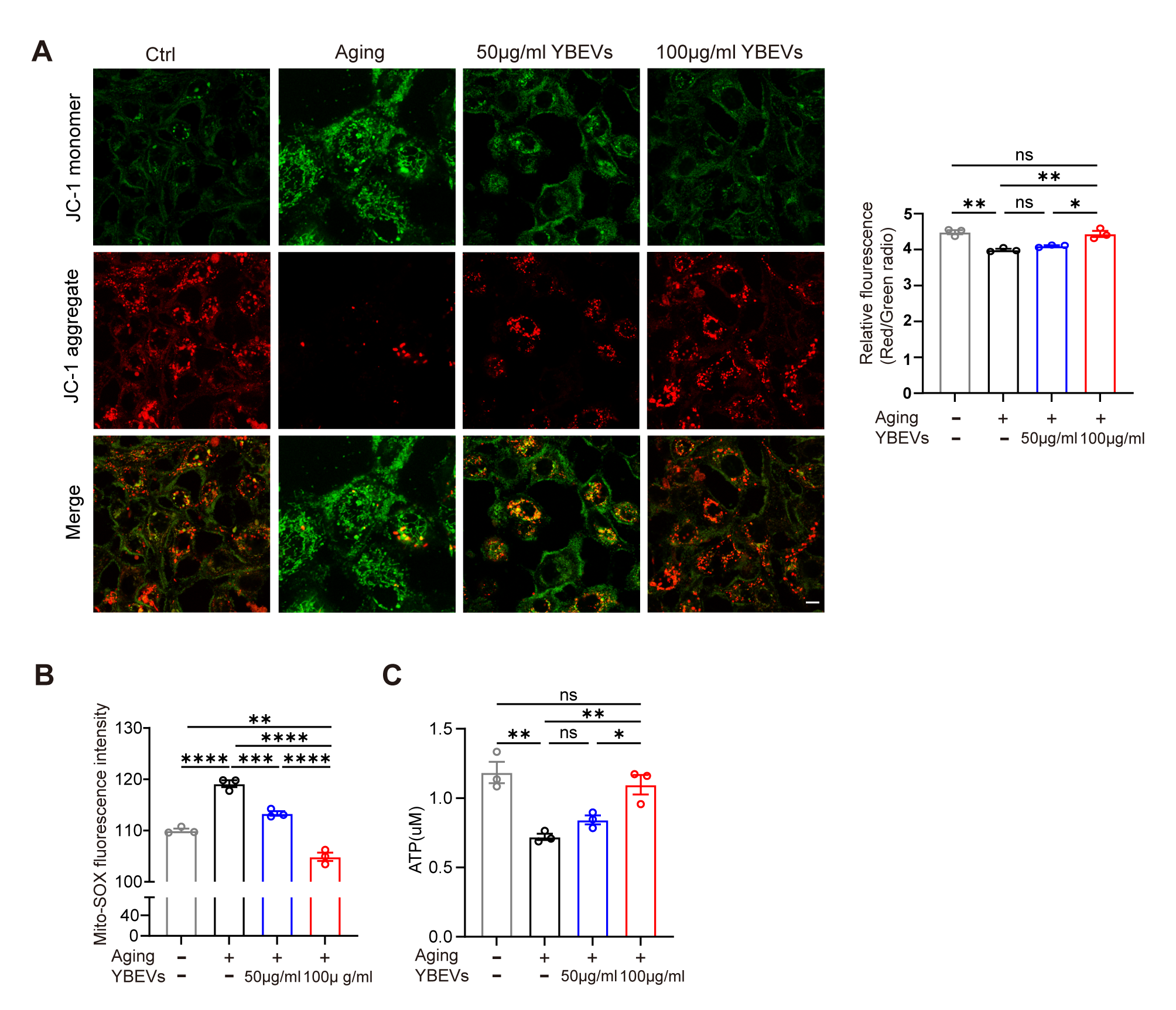

Supplement: Supplementary 1 — Figs. S1 to S6 Table S1 [file research.0644.f1.zip › S6.tif]
